# Virtual-patient nonlinear mixed-effects modelling for data-driven optimisation of single-time-point dosimetry: a proof-of-concept study in [^177^Lu]Lu-PSMA-617 therapy

**DOI:** 10.1186/s40658-026-00933-w

**Published:** 2026-08-30

**Authors:** Yeni Pertiwi, Indra Budiansah, Jaja Muhamad Jabar, Muchtaridi Muchtaridi, Freddy Haryanto, Idam Arif, Deni Hardiansyah, Gerhard Glatting

**Affiliations:** 1 Nuclear Physics and Biophysics Research Group, Physics Department, Faculty of Mathematics and Science, Institut Teknologi Bandung, Bandung, Indonesia; 2 Department of Medical Electronics Engineering Technology, Faculty of Health Technology, Al Insyirah Institute of Health and Technology, Pekanbaru, Indonesia; 3 Medical Physics and Biophysics, Physics Department, Faculty of Mathematics and Science Physics, Universitas Indonesia, Depok, Indonesia; 4 Diploma Program in Optometry, School of Health Sciences (STIKes), Dharma Husada Bandung, Bandung, 40282 Indonesia; 5 Department of Pharmaceutical Analysis and Medicinal Chemistry, Faculty of Pharmacy, Universitas Padjadjaran, Sumedang, West Java Indonesia; 6 Medical Radiation Physics, Department of Nuclear Medicine, Ulm University, Albert-Einstein-Allee 23, 89081 Ulm, Germany

**Keywords:** Virtual patients, Single-time-point dosimetry, Time-integrated activity coefficient, Nonlinear mixed-effects model, [¹⁷⁷Lu]Lu-PSMA-617, Radioligand therapy, Kidneys

## Abstract

**Background:**

Robust single-time-point (STP) dosimetry critically depends on selecting an imaging time that yields reliable estimates of the time-integrated activity coefficient (TIAC). This study developed and evaluated a virtual-patient nonlinear mixed-effects modelling (NLMEM) framework to optimise STP imaging schedules for renal TIAC estimation, using [¹⁷⁷Lu]Lu-PSMA-617 therapy as a proof of concept.

**Methods:**

A previously published NLMEM (sum-of-exponentials structure and parameter distributions) describing renal [¹⁷⁷Lu]Lu-PSMA-617 biokinetics in 63 patients served as the generative model [1]. Based on fixed- and random-effects parameters, 500 virtual patients (VPs) were sampled, and reference TIAC values were computed analytically (rTIAC). For each candidate imaging time, renal activity measurements were simulated by applying proportional noise (7.9%). Estimated STP TIAC values (sTIAC) were obtained via leave-one-out cross-validation and compared with rTIAC to quantify population-level error metrics using mean absolute percentage error (MAPE) and root-mean-square error (RMSE). Time-dependent MAPE–time and RMSE–time profiles were modelled using polynomial fits selected by predefined goodness-of-fit criteria to identify time windows with low and stable errors. Individual-level robustness was assessed by quantifying extreme patient-specific relative deviations (RD) using thresholds of 10%, 20%, and 30%.

**Results:**

MAPE- and RMSE-versus-time profiles were concordant and showed a pronounced minimum around 50 h post-injection. Among the investigated imaging time points, the lowest observed population-level error metrics occurred at 48 h, whereas polynomial modelling predicted an optimum centred at approximately 50 h (about 33–67 h). An additional VP-NLMEM evaluation at 50 h demonstrated comparable population-level performance and the lowest frequency of |RD| >30%, while the minima for the 10% and 20% thresholds occurred at nearby imaging times.

**Conclusion:**

The proposed virtual-patient NLMEM framework enables data-driven optimisation of STP imaging by identifying robust time windows that minimise both population-level error and extreme individual deviations in renal TIAC estimation for [¹⁷⁷Lu]Lu-PSMA-617 therapy. The approach appears transferable to STP dosimetry optimisation for other radiopharmaceuticals.

**Supplementary Information:**

The online version contains supplementary material available at https://doi.org/10.1186/s40658-026-00933-w.

## Introduction

Molecular radiotherapy (MRT) with [^177^Lu]Lu-PSMA-617 has been shown to be an effective treatment for metastatic castration-resistant prostate cancer (mCRPC). Clinical studies reported that this therapy led to a significant decline in prostate-specific antigen (PSA) (≥ 50% in 57% of patients) and improved progression-free survival, ranging from 3 to 13.7 months [2, 3]. This therapy provides a 3–6 times higher absorbed dose to the tumour than to the kidney and salivary glands [4, 5]. Although [^177^Lu]Lu-PSMA-617 therapy is effective, several organs, such as the kidneys, salivary glands, and lacrimal glands, are considered at risk of unintended radiation exposure [4, 6]. Current EANM/SNMMI recommendations also identify bone marrow as an organ requiring dosimetric consideration during [^177^Lu]Lu-PSMA-617 therapy [7, 8]. Haematological toxicity is also recognised as an important clinical consideration during this therapy [9, 10]. Therefore, robust personalised dosimetry is essential to optimise treatment planning by delivering effective doses to tumours and minimising radiation exposure to organs at risk [11]. In addition to treatment planning, personalised dosimetry is also essential for dose verification and for establishing dose–effect relationships that support evidence-based optimisation of molecular radiotherapy [8].

Personalised dosimetry plays a critical role in optimising MRT by enabling patient-specific treatment planning and risk assessment. However, its widespread implementation remains challenging due to the logistical and operational burden of acquiring multiple post-therapy imaging time points. These repeated imaging sessions not only increase patient discomfort and radiation exposure but also impose significant strain on clinical resources and workflow [12]. To address these challenges, Single-Time-Point (STP) dosimetry has been proposed as a practical alternative to conventional multi-time-point dosimetry protocols. The STP approach aims to estimate time-integrated activity coefficients (TIACs) from a single imaging acquisition, substantially simplifying imaging data collection while preserving acceptable dosimetric performance. Several studies have demonstrated the feasibility of STP dosimetry in molecular radiotherapy. Hardiansyah et al. [1] demonstrated that renal STP dosimetry using a nonlinear mixed-effects modelling framework achieved the lowest dosimetric error at approximately 42.6 h post-injection. Similarly, Brosch-Lenz et al. [13] demonstrated that image-based STP dosimetry for [¹⁷⁷Lu]Lu-PSMA-617 provided acceptable agreement with reference dosimetry and could facilitate routine clinical implementation.

Recently, Gomes et al. [14] trained a machine-learning model to predict organ-specific effective half-lives from pretherapy imaging and clinical data to enable STP dosimetry. They proposed a maximum mean absolute percentage error (MAPE) of 27% as an acceptable accuracy threshold for STP dosimetry and showed that, based on population-level accuracy metric MAPE, STP dosimetry could theoretically be performed over a wide range of imaging times, including very early acquisitions. However, population-level accuracy metrics such as MAPE do not capture the full error distribution or the occurrence of extreme deviations at the individual-patient level. Indeed, Gomes et al. [14] reported individual relative deviations of up to 60% at early imaging times, and Hardiansyah et al. [1] similarly demonstrated that even at the previously identified optimal time point, i.e., 42.6 h post-injection, individual deviations could still exceed 40% for [¹⁷⁷Lu]Lu-PSMA-617. These findings highlight that population-level accuracy analysis alone is insufficient to ensure the clinical reliability of STP dosimetry for patient-specific decision making. Consequently, identifying an optimal imaging time point for STP dosimetry remains highly desirable. Still, it requires joint evaluation of population-level accuracy metrics (e.g., MAPE and root-mean-squared errors (RMSE)) and individual-level performance, including extreme individual-level deviations. In this setting, optimality is inseparable from accuracy robustness, as a time point with acceptable mean accuracy but frequent large individual errors may be clinically unacceptable. Therefore, clinically robust STP protocols must explicitly integrate both population- and individual-level accuracy metrics.

In practice, however, conducting systematic evaluations to identify the most favourable imaging time point for STP dosimetry is challenging when relying solely on empirical patient imaging data. Clinical patient imaging datasets are typically limited to three to five acquisition time points due to logistical constraints and radiation exposure considerations [13, 15–21]. As a result, the identification of “optimal” STP imaging time is inherently constrained by the availability of measured time points rather than being identified through a continuous assessment across the full biokinetic time course. To overcome these limitations, virtual-patient simulations provide a powerful framework for STP dosimetry optimisation. By leveraging population-informed biokinetic models, virtual patients enable continuous evaluation of dosimetric accuracy across various time domains, independent of clinically constrained acquisition schedules. Importantly, this approach allows simultaneous assessment of population-level accuracy metrics and individual-level extreme deviations at every potential imaging time point, facilitating the identification of truly robust STP imaging windows. Such capability is particularly critical for new radiopharmaceuticals or early clinical adoption scenarios, where patient data are sparse, site-specific protocols vary, and empirical optimisation based on large datasets is not feasible.

Therefore, in this study, we develop a virtual-patient nonlinear mixed-effects modelling (VP-NLMEM) framework to address these challenges. This framework enables systematic optimisation of STP imaging time by quantifying both population-level accuracy and the robustness of individual-level errors across candidate imaging times. As a proof of concept, we apply the method to [¹⁷⁷Lu]Lu-PSMA-617 therapy specifically for renal dosimetry and demonstrate its ability to identify clinically robust STP dosimetry windows under realistic constraints of limited sampling.

## Materials and methods

### Virtual patients

The VP-NLMEM framework was started by creating simulated biokinetic data. In this study, biokinetic data of [^177^Lu]Lu-PSMA-617 in the kidney were simulated using the Sums-of-Exponentials Function (SOEF) within the NLMEM framework (Eq. 1). This SOEF had been previously identified as the optimal model for representing the biokinetics of [^177^Lu]Lu-PSMA-617 in the kidney of 63 patients with mCRPC [1]. Its suitability as a reference model for renal dosimetry has subsequently been supported by recent comparative evaluation [22]. Therefore, this SOEF model was employed here to generate virtual patients (VPs):

1 $$\:{f}_{6}\left(t\right)={A}_{1}{e}^{-\left({\lambda\:}_{1}+{\lambda\:}_{phys}\right)t}+{A}_{2}{e}^{-({\lambda\:}_{2}+{\lambda\:}_{phys})t}-{A}_{3}{e}^{-\left({\lambda\:}_{3}+{\lambda\:}_{phys}\right)t}-({A}_{1}{+{A}_{2}-{A}_{3})e}^{-\left({\lambda\:}_{bc}+{\lambda\:}_{phys}\right)t}\:\:\:\:\:\:\:$$

where $\:{f}_{6}\left(t\right)$ is the SOEF with six adjusted parameters that models the biological and physical kinetics of radiopharmaceutical uptake and clearance, the $\:{A}_{j}$ (j = 1,2,3) represent the prefactors of the function with values ≥ 0, the $\:{\lambda\:}_{j}$ (j = 1,2,3) describe the biological clearance or uptake rates of the radiopharmaceutical in the kidneys with values ≥ 0, $\:{\lambda\:}_{phys}$ denotes the physical decay constant of ^177^Lu, calculated as (ln(2)/half-life) with a half-life of 6.6443 days [23], $\:{\lambda\:}_{bc}$ represents the blood circulation rate, with half-life of 1 min (ln(2)/1 min) [1, 24, 25].

Parameter distributions, defined by the fixed-effects and random-effects estimates reported in the literature (Table 2 in [1]), were used to simulate individual-level parameters consistent with the reported population variability. For each VP, random effects were sampled from a normal distribution N(0, $\:{\sigma\:}_{i}^{2}$), where $\:{\sigma\:}_{i}^{2}$ corresponds to the variance of the respective random-effects parameter. The individual parameters ($\:{P}_{i}$) for each VP were then calculated as follows:

2 $$ p_{{i,j}} = TVP_{j} \times \exp (ETA_{i} ) $$

3 $$ETA_{i} = N(0,\sigma _{i}^{2} ) $$

where $\:{P}_{i,j}$ is the j-th estimated parameter for the *i*-th individual, $\:TV{P}_{j}$ represents the fixed-effects, and $\:{\:ETA}_{i}$ represents the random effects of each parameter in each VP.

## Study workflow

**Fig. 1:**
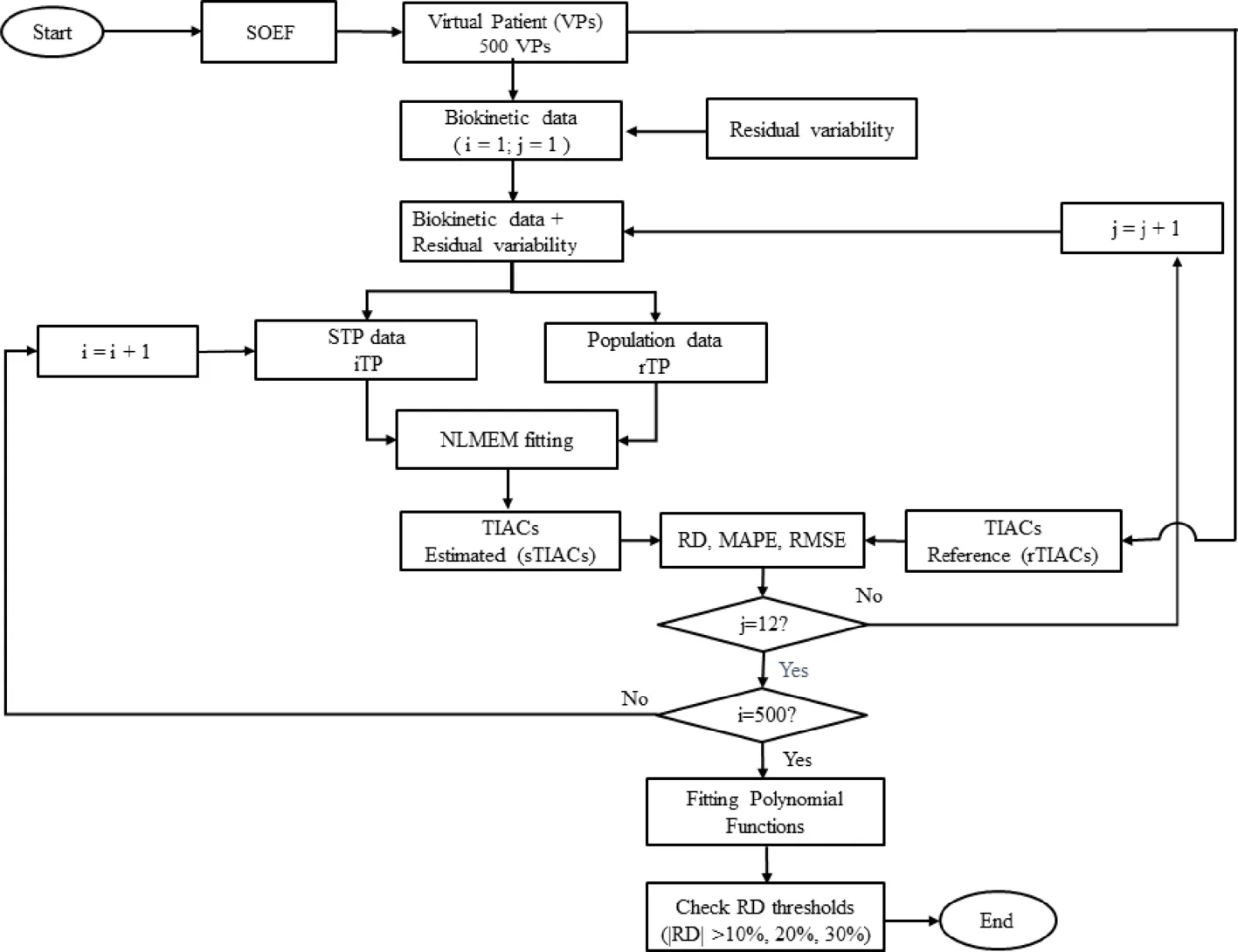
Study workflow. The SOEF model (Eq. 1) was used to generate 500 virtual patients (VPs), and reference TIACs (rTIACs) were calculated analytically. Biokinetic data were simulated with added intra-individual variability. Simulated biokinetic data at twelve investigational time points (iTPs) were evaluated for STP dosimetry, whereas simulated biokinetic data at five reference time points (rTPs) [1] were used as the population data for NLMEM. Population biokinetic data at the rTPs were simultaneously fitted with the STP biokinetic data at each iTP data using NLMEM. STP-estimated TIACs (sTIACs) were compared with rTIACs using RD, RMSE, and MAPE. Fitting of RMSE-time and MAPE-time trends identified the optimal STP imaging time point. Absolute RDs exceeding 10%, 20%, and 30% were used for patient-level analysis. *i* = virtual patient index; *j* = investigational time point

Figure 1 shows the VP-NLMEM workflow of this study. A total of 500 VPs were generated using the SOEF Eq. 1. This number was chosen to balance clinical variability representation and computational efficiency, consistent with previous studies on STP dosimetry using ¹⁷⁷Lu-based therapies [26]. Reference Time-Integrated Activity (rTIAC) values were then calculated analytically from the individual parameter sets generated using Eq. (1).

Biokinetic data were generated with intra-individual variability for each VP at 12 investigational time points (iTPs) for STP dosimetry: 18.7 h, 24 h, 30 h, 36 h, 42.6 h, 48 h, 54 h, 60 h, 66.3 h, 72 h, 78 h, 84 h, and 5 reference time points (rTPs) used as population biokinetic data for NLMEM: 1.8 h, 18.7 h, 42.6 h, 66.3 h, and 160.3 h [1]. The rTPs were selected based on commonly used clinical imaging measurement times of [^177^Lu]Lu-PSMA-617 kinetics in the literature [1, 27], and among these, 42.6 h after injection yielded the lowest RMSE for renal dosimetry [1]. To examine whether this time was truly optimal for STP dosimetry, the iTPs were systematically investigated around 42.6 h after injection (earlier and later times at 6-h intervals), thereby enabling a more refined evaluation of the optimal imaging time.

To account for measurement variability in the simulated biokinetic data of [^177^Lu]Lu-PSMA-617, a proportional residual error (i.e., measurement errors, residual variability) of 7.9% [1] was applied to the simulated biokinetic data generated from the SOEF model. This variability was modelled using a proportional error model (Eq. 4):

4 $$ y_{{i,j}} = f_{{i,j}} + (f_{{i,j}} \times a \times \varepsilon ) $$

where $\:{y}_{i,j\:}$ is the biokinetic data at TP j and VP * i*, $\:{f}_{i,j}$ is the simulated biokinetic data from the SOEF function, * a* is the measurement error represented by the residual variability of the data, and $\:\varepsilon$ is a normally distributed random variable with mean 0 and variance 1.

Furthermore, to estimate the Time-Integrated Activity (sTIACs), a leave-one-out cross-validation strategy was employed. In each iteration, one virtual patient was sequentially treated as the STP subject, whereas the remaining virtual patients constituted the population dataset for simultaneous NLMEM fitting [28]. The fitting processes were performed using NONMEM software (version 7.5.1; ICON Development Solutions, Ellicott City, MD, USA). R software (version 4.4.1) was used to generate virtual patient parameter datasets with intra-individual variability, automate the creation and execution of NONMEM control files, extract model outputs, and evaluate RD, MAPE, and RMSE. The accuracy of the sTIACs was assessed using the RD, MAPE, and RMSE with rTIACs as the reference [29, 30]:

5 $$\:{RD}_{i,j}=\:\frac{{sTIAC}_{i,j}-{rTIAC}_{i,j}\:}{{rTIA}_{i,j}}$$

6 $$\:{\:\:\:\:\:MAPE}_{j}=\:\frac{1}{N}\:{\sum\:}_{i=1}^{N}\left|\frac{{sTIAC}_{i,j}-{rTIAC}_{i,j}\:}{{rTIA}_{i,j}}\right|$$

7 $$\:{RMSE}_{j}=\:\sqrt{\frac{1}{N}{\sum\:}_{i=1}^{N}{RD}_{i,j}^{2}}$$

where *N* denotes the total number of VPs, the * i*-th virtual patient, and the * j*-th time point, respectively. The uncertainties of RD ($\:{U}_{RD\:})$, MAPE ($\:{U}_{MAPE\:})$, and RMSE ($\:{U}_{RMSE})$ were calculated using error propagation [28, 31]. Details are presented in the Supplemental file (S1-S5).

Subsequently, fitting was applied to the MAPE-time and RMSE-time data to determine the optimal time point for STP dosimetry based on the observed trends in these metrics. Three polynomial models with increasing complexity were employed to describe the variation of MAPE and RMSE. The minimum location of the fitted curves was defined by parameter b. All polynomial equations and detailed model specifications are provided in the Supplementary Material (Eqs. S6–S8).

Each model parameter was estimated using nonlinear least-squares fitting implemented in SAAM II (version 2.1; Software Applications for Kinetic Analysis, University of Washington and The Epsilon Group, 2011). A relative variance and proportional error model was used.

The selection of the polynomial function was based on: (i) visual inspection of the fitted curves, and (ii) quantitative evaluation of the model fit using the Goodness-of-Fit (GoF) criterion, defined by the coefficient of variation (CV) of < 50% [29, 32]. The selected polynomial function was subsequently used to define the optimal time window for STP dosimetry, based on a predefined accuracy criterion of MAPE < 10%. In addition, NLMEM STP fitting was performed at time points with the lowest MAPE and RMSE identified from the selected polynomial function. This approach provides a systematic framework for identifying the optimal sampling time by analysing the temporal behaviour of the performance at the population level.

Individual-level analiysis was evaluated by assessing individual relative deviation (RD) values at each time point. Absolute RD thresholds of 10%, 20%, and 30% were applied to identify cases exceeding predefined accuracy limits. Patients with RD values above these thresholds were classified as exhibiting extreme deviations. This approach enabled a systematic assessment of sTIACs at the individual patient level.

## Results

**Fig. 2:**
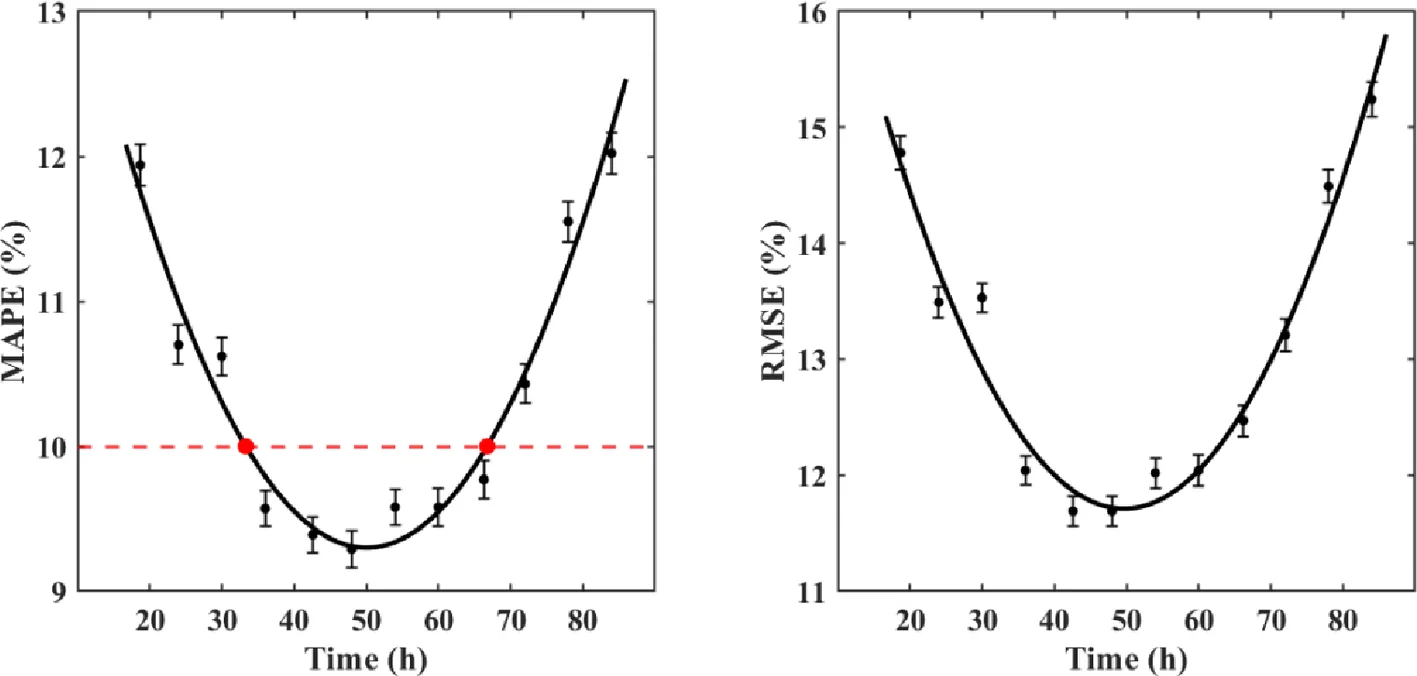
Polynomial fitting of MAPE and RMSE curves based on Equation (S6). Both curves exhibit an upward-opening parabolic shape, with minimum RMSE and MAPE values observed at approximately 50 h after injection

The STP simulation results across iTP are summarised in Table 1. The lowest MAPE and RMSE values of 9.3% ± 0.6% and 11.7% ± 0.6%, respectively, were observed at 48 h based on the NLMEM fit. At earlier time points, both MAPE and RMSE values were generally higher, and they increased again after 54 h. Figure 2 (S6) shows that polynomial model 1 consistently provides the best description of the MAPE-time and RMSE-time trends. This observation is supported by the GoF evaluation, where model 1 (S6) was the only function that satisfied the GoF criterion (CV < 50%) for all fitted parameters, resulting in maximum CV values of 7.42% for MAPE and 7.20% for RMSE (Table S2). Furthermore, polynomial function fitting subsequently predicted the minima of the MAPE and RMSE curves at approximately 50 h post-injection (Table S3), which was further evaluated by an additional VP-NLMEM simulation at the predicted optimum.

**Table 1:** Summary of RD, MAPE, and RMSE from model evaluation across iTP

Time point	RD mean ± $\:{\boldsymbol{U}}_{\boldsymbol{R}\boldsymbol{D}\:}$	MAPE ± $\:{\boldsymbol{U}}_{\boldsymbol{M}\boldsymbol{A}\boldsymbol{P}\boldsymbol{E}\:}$	RMSE ± $\:{\boldsymbol{U}}_{\boldsymbol{R}\boldsymbol{M}\boldsymbol{S}\boldsymbol{E}}$
18.7	− 2.8 ± 0.7	11.9 ± 0.7	14.8 ± 0.7
24	− 3.5 ± 0.7	10.7 ± 0.7	13.5 ± 0.7
30	− 2.8 ± 0.6	10.9 ± 0.6	13.4 ± 0.6
36	− 2.1 ± 0.6	9.6 ± 0.6	12.0 ± 0.6
42.6	− 1.4 ± 0.6	9.4 ± 0.6	11.7 ± 0.6
48	− 0.9 ± 0.6	9.3 ± 0.6	11.7 ± 0.6
50*	− 0.1 ± 0.6	9.5 ± 0.6	11.9 ± 0.6
54	0.4 ± 0.6	9.6 ± 0.6	12.0 ± 0.6
60	0.4 ± 0.6	9.6 ± 0.6	12.0 ± 0.7
66.2	0.5 ± 0.7	9.8 ± 0.7	12.5 ± 0.7
72	1.2 ± 0.7	10.4 ± 0.7	13.2 ± 0.7
78	1.5 ± 0.7	11.5 ± 0.7	14.5 ± 0.7
84	1.4 ± 0.7	12.0 ± 0.7	15.2 ± 0.7

*The model-derived optimum at 50 h was evaluated as an additional candidate time point for patient-level RD-threshold analysis

**Fig. 3:**
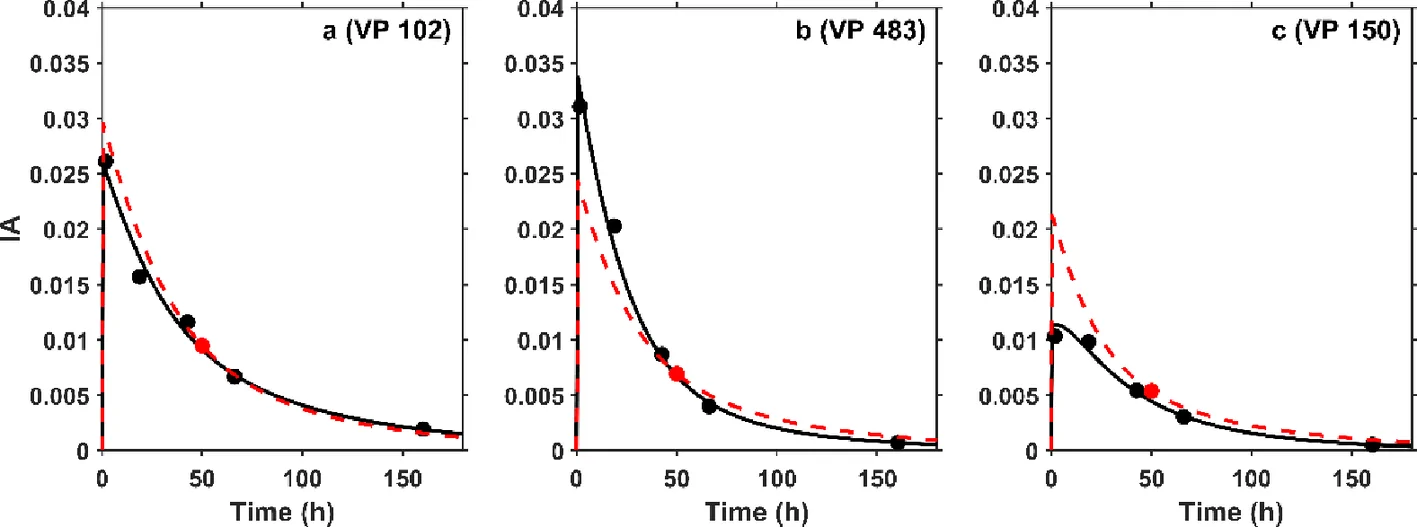
Comparison of model-estimated time-activity curves (TACs) for selected virtual patients (VPs) with (**a**) minimum (VP 102), (**b**) median (VP 483), and (**c**) maximum (VP 150) relative deviation (RD). Solid lines represent TACs analytically derived from the full set of simulated parameters (rTP), and dashed red lines represent TACs predicted using a single iTP at 50 h after injection

Figure 3 shows a comparison between time-activity curves from reference and STP, consisting of (a) the minimum (VP 102) RD, (b) the median (VP 483), and (c) the maximum RD (VP 150). The comparison demonstrates that for patients with minimum RD, the STP-predicted kinetics closely match the reference kinetics. The median case shows a moderate level, while patients with higher RD exhibit noticeable discrepancies, particularly at early and late time points.

**Fig. 4:**
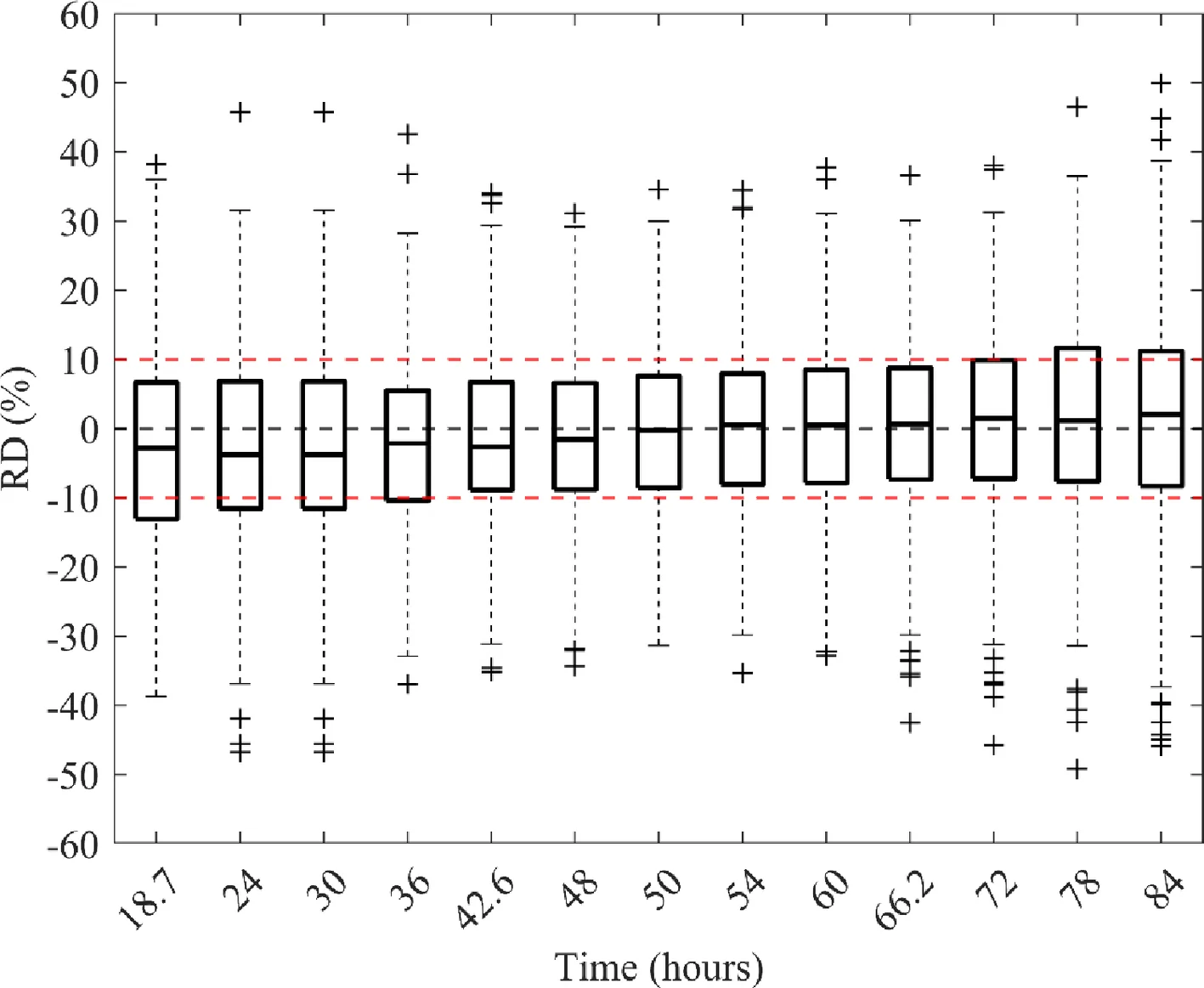
Distribution of relative deviations (RD) across 12 investigated imaging time points (18.7–84 h post-injection) and for the additional model-derived 50 h time point. The 50 h time point was obtained from polynomial fitting and subsequently evaluated by an additional VP-NLMEM simulation to assess the individual-level RD distribution. Boxplots display the median RD and statistical outliers (values beyond 1.5×IQR). Notably fewer outliers were observed at 50 h after injection, indicating reduced interpatient variability at this time point

Figure 4 shows the distribution of RD values at 12 time points (18.7–84 h) post-injection together with the additional evaluation at the model-derived optimum (50 h). As summarised in Table 2, the proportions of patients exceeding the predefined |RD| thresholds varied according to the selected threshold. The lowest proportion of |RD| >10% occurred at 48 h, whereas the lowest proportion of |RD| >20% was observed at 36 h and 42.6 h. The additional 50 h evaluation yielded the lowest proportion of |RD| >30%. Collectively, the 33–67 h interval showed consistently low exceedance rates across all thresholds. Conversely, the earliest (18.7 h) and latest (84 h) imaging time points exhibited substantially higher proportions of large deviations and a greater frequency of extreme RD values.

**Table 2:** Percentage of patients (out of *N* = 500) with individual absolute RD values exceeding the 10%, 20%, and 30% thresholds at each time point

Time point (h)	RD values exceeding threshold (%)		
	10%	20%	30%
18.7	51.8	17.4	4.4
24	47.0	11.4	2.0
30	47.0	11.4	2.0
36	42.8	8.4	1.0
42.6	38.8	8.4	1.4
48	38.2	9.4	1.0
50*	40.8	9.6	0.4
54	41.4	10.4	1.0
60	41.2	9.6	1.6
66.2	41.0	11.4	1.6
72	43.2	13.0	2.2
78	49.4	16.6	3.4
84	50.4	17.4	5.0

*The model-derived optimum at 50 h was evaluated as an additional candidate time point for patient-level RD-threshold analysis

## Discussion

In this work, we present a virtual-patient nonlinear mixed-effects modelling (NLMEM) framework designed to systematically optimise single-time-point (STP) imaging in molecular radiotherapy. By enabling joint evaluation of population-level accuracy and individual-level error robustness across the full biokinetic time course, this approach addresses key limitations of empirically derived STP protocols that rely on sparse clinical data. Using [^177^Lu]Lu-PSMA-617 therapy as a proof of concept, our results demonstrate the feasibility of identifying model-derived robust STP imaging windows under realistic conditions of data limitation.

Across the investigated time range, optimal STP performance was identified within a post-injection interval of approximately 33–67 h, defined by the intersection of the selected polynomial MAPE–time curve with the 10% accuracy threshold. Among the investigated imaging time points, the lowest observed MAPE and RMSE occurred at 48 h, whereas polynomial fitting predicted an optimum at approximately 50 h. Within the 33–67 h window, |RD| > 30% values remained consistently below 2% (Table 2), indicating that the population-level accuracy criterion aligned with a reduction in extreme patient-level deviations.

Analysis of RD distributions further demonstrated that the 33–67 h interval provided a favourable balance between population-level error metrics and individual-level deviations. Although the minima of the individual RD thresholds occurred at different imaging times, the additional evaluation at the model-predicted optimum (50 h) yielded the lowest proportion of |RD| >30%. Median RD values shifted from underestimation at early imaging times to overestimation at later times, with a near-zero crossing within the 33–67 h window (Fig. 4; Table 1). In contrast, early (18.7 h) and late (84 h) acquisitions showed a substantially higher frequency and magnitude of extreme deviations, despite acceptable population-level MAPE (9.3%–12%) and RMSE values. These findings highlight an important limitation of relying solely on mean accuracy metrics: acceptable population-level performance does not guarantee reliable patient-level dosimetry.

Figure 3 illustrates the remaining interindividual variability at the optimal 50 h time point, showing representative virtual patients with minimal, median, and maximal deviations. Even at the population-optimal time for the STP dosimetry, patient-specific errors varied, underscoring the importance of integrating individual-level error assessment into STP optimisation.

Our findings are consistent with previous studies demonstrating that STP dosimetry is feasible when the imaging time is appropriately selected. For [^177^Lu]Lu-PSMA-targeted therapy, Hardiansyah et al. [1], Brosch-Lenz et al. [13], and Burkett [33] independently reported that imaging at approximately 2 days post-injection provided favourable agreement with reference multiple-time-point dosimetry for renal dosimetry. The present VP-NLMEM framework similarly identified an optimal STP imaging time of approximately 50 h post-injection, supporting the same general time range reported in previous studies. Unlike previous studies, however, the proposed framework extends the analysis from discrete clinical sampling times to continuous evaluation across the investigated post-injection time range. Similar model-informed approaches have also been explored for other radiopharmaceuticals. For example, Wang et al. [26] showed that data-driven prediction models reduced the sensitivity of STP dosimetry to imaging time selection for [^177^Lu]Lu-DOTATATE. Collectively, these studies support the use of model-informed approaches for optimising STP dosimetry. The proposed VP-NLMEM framework further identifies a model-derived robust imaging window (33–67 h) through continuous virtual-patient simulations and integrates both population-level performance and individual-level robustness into the optimisation process. Beyond confirming existing findings, this capability is particularly relevant for emerging radiopharmaceuticals and early clinical adoption scenarios, where patient datasets are limited, imaging schedules vary across centres, and empirical optimisation based solely on clinical observations is not feasible. In such settings, a VP-NLMEM framework can be constructed from the available data, even when it is sparsely sampled. When necessary, physiologically plausible assumptions or parameter values derived from relevant literature can be incorporated to ensure biologic consistency and model stability.

Once established, the VP-NLMEM framework enables systematic simulation of STP dosimetry performance across a continuous range of candidate imaging time points. Performance metrics such as MAPE, RMSE, and the distributions of individual relative deviations can be evaluated to identify a clinically acceptable time window. The predefined accuracy criterion may follow thresholds such as MAPE < 10%, as applied in this study, or alternative limits determined in collaboration with treating physicians based on clinical priorities and acceptable risk margins. Importantly, this model-informed strategy allows subsequent confirmatory STP studies to focus specifically on the predicted optimal measurement range, rather than exploring multiple empiric time points. Such a targeted approach enhances study efficiency, reduces patient burden, and supports faster, evidence-informed standardisation of STP dosimetry protocols in early clinical implementation.

In clinical practice, however, implementation of the model-derived STP imaging time may be influenced by institution-specific patient-release criteria, local radiation-protection regulations, scanner availability, and the feasibility of patient return. Consequently, the predicted imaging time should not be interpreted as a mandatory imaging time for all centres. Instead, the proposed VP-NLMEM framework provides population-level and individual-level error profiles across candidate imaging times, allowing clinicians to select an imaging schedule that is compatible with local clinical constraints while remaining fully informed about the expected dosimetric performance and associated uncertainty.

The optimal STP imaging time is expected to differ among the kidneys, salivary glands, lacrimal glands, bone marrow, and tumours because these tissues exhibit distinct biokinetic characteristics. Consequently, a single imaging time selected for routine clinical implementation is likely to represent a practical compromise rather than the true optimum for every organ and tissue. One possible strategy to address this challenge is to assign different weights to organs or tumours according to the intended clinical objective. This approach enables selection of a clinically appropriate compromise imaging time [28]. Such an evaluation can simultaneously consider both population-level accuracy and the robustness of individual-level errors. Within this framework, the proposed VP-NLMEM method can be used to evaluate such weighted objectives across candidate imaging times, thereby supporting clinically informed decision-making.

Limitations of this study should be acknowledged. First, this analysis was based on a single patient cohort and requires validation in independent populations and across multiple centers to establish generalisability. Second, the present evaluation focused on renal dosimetry. Future work extending the VP-NLMEM framework to other organs at risk - such as the salivary glands, lacrimal glands, bone marrow, and tumour is warranted. Finally, while the modelling results are encouraging, prospective clinical studies are needed to assess feasibility and reproducibility of VP-NLMEM.

## Conclusion

In this study, we developed a VP-NLMEM framework for data-driven optimisation of STP dosimetry by jointly evaluating population- and individual-level relative deviation across the full biokinetic time course. In a proof-of-concept application to renal dosimetry in [^177^Lu]Lu-PSMA-617 therapy, the framework identified a model-predicted optimal STP imaging time of approximately 50 h post-injection, consistent with previously reported time ranges for renal dosimetry - providing additional support for the proposed VP-NLMEM framework. More broadly, this strategy provides a structured approach for emerging radiopharmaceuticals and early clinical implementation, where datasets may be sparse, by enabling simulation-based identification of clinically acceptable STP time windows and guiding focused confirmatory studies toward efficient and standardised protocol adoption.

## Supplementary Information

Below is the link to the electronic supplementary material.


Supplementary Material


## Data Availability

The data used are available from the corresponding author upon reasonable request.

## Abbreviations

sTIACs STP: estimated time-integrated activity coefficients
FSD: Fractional standard deviation
iTP: Investigational time point
MAPE: Mean-absolute-percentage error
NLMEM: Nonlinear mixed-effect model
PBMS: Population-based model selection
RD: Relative deviation
RMSE: Root-mean-square error
rTIACs: Reference time-integrated activity coefficients
rTP: Reference time point
SOEF: Sum-of-the-exponential function
STP: Single time point
TAC: Time-activity curve
TIACs: Time-integrated activity coefficients
TP: Time point
