# Supplementary material for: Virtual-patient nonlinear mixed-effects modelling for data-driven optimisation of single-time-point dosimetry: a proof-of-concept study in [177Lu]Lu-PSMA-617 therapy
Source: EJNMMI Phys. 2026 Aug 30;13:87. doi: 10.1186/s40658-026-00933-w (PMC13529636; doi:10.1186/s40658-026-00933-w)
Supplement: Supplementary file 1 [file 40658_2026_933_MOESM1_ESM.docx]

**Supplemental File**

Virtual-Patient Nonlinear Mixed-Effects Modelling for Data-Driven Optimisation of Single-Time-Point Dosimetry: A Proof-of-Concept Study in [^177^Lu]Lu-PSMA-617 Therapy

Yeni Pertiwi^1, 2^, Indra Budiansah^3^, Jaja Muhamad Jabar^1, 4^, Muchtaridi Muchtaridi^5^, Freddy Haryanto^1^, Idam Arif^1^, Deni Hardiansyah^3, 6*$^, Gerhard Glatting**^6^**^$^

*^1^Nuclear Physics and Biophysics Research Group, Physics Department, Faculty of Mathematics and Science, Institut Teknologi Bandung, Bandung, Indonesia*

*^2^ Department of Medical Electronics Engineering Technology, Faculty of Health Technology, Al Insyirah Institute of Health and Technology, Pekanbaru, Indonesia*

*^3^Medical Physics and Biophysics, Physics Department, Faculty of Mathematics and Science Physics, Universitas Indonesia, Depok, Indonesia*

*^4^Diploma Program in Optometry, School of Health Sciences (STIKes) Dharma Husada Bandung, Bandung 40282, Indonesia*

*^5^Department of Pharmaceutical Analysis and Medicinal Chemistry, Faculty of Pharmacy, Universitas Padjadjaran, Sumedang, West Java, Indonesia*

*^6^Medical Radiation Physics, Department of Nuclear Medicine, Ulm University, Ulm, Germany*

^$^Shared senior authorship

*Corresponding Author:

Dr. sc. hum. Deni Hardiansyah

1. Ulm University, Albert-Einstein-Allee 23, 89081 Ulm, Germany
2. Physics Department, Faculty of Mathematics and Natural Sciences (FMIPA),, Universitas Indonesia, Depok, 16424, Indonesia , Email (University): [denihardiansyah@sci.ui.ac.id](mailto:denihardiansyah@sci.ui.ac.id)

**Part A. Uncertainty Propagation in RD, RMSE, and MAPE Calculations**

The following section presents the uncertainty propagation formulas used to calculate the uncertainties of RD, RMSE, and MAPE. These equations are derived from general principles of error propagation [1,2] and are applied to both individual and population parameters.

$U_{{RD}_{i,j}} = \sqrt{\left( \frac{U_{{sTIAC}_{i,j}}}{{rTIA}_{i,j}} \right)^{2}+ \left( \frac{{sTIA}_{i,j} . U_{{rTIAC}_{i,j}}}{{{rTIA}_{i,j}}^{2}} \right)^{2}}$	(S1)

$U_{{RMSE}_{i,j}}= \sqrt{\left( \frac{{{RD}_{i,j}}_{mean}}{{RMSE}_{i,j}} U_{{{RD}_{i,TP}}_{mean}} \right)^{2}+ \left( \frac{{SD}_{{RD}_{i,j}}}{{RMSE}_{i,j}} U_{{SD}_{{RD}_{i,j}}} \right)^{2}}$	(S2)

$U_{{MAPE}_{i,j}}= \sqrt{\sum_{j=1}^{n} \left[ \left( \frac{U_{{sTIAC}_{i,j}}}{n . {rTIAC}_{i,j}} \right)^{2}+ \left( \frac{{sTIAC}_{i,j}. U_{{rTIAC}_{i,j}}}{n . {{rTIAC}_{i,j}}^{2}} \right)^{2} \right]}$	(S3)

With:

$U_{{{RD}_{i,j}}_{mean}}= \sqrt{\sum_{i=1}^{n} \left( \frac{{U_{RD}}_{i,j}}{n} \right)^{2}}$	(S4)

$U_{{SD}_{RD}} = \sqrt{\sum_{i=1}^{n} \left( \frac{{RD}_{i,j}- {{RD}_{i,j}}_{mean}}{\left( n-1 \right){SD}_{{RD}_{i,j}}} . U_{{RD}_{i,j}} \right)^{2}}$	(S5)

The uncertainty of RD ($U_{RD})$is calculated from the value of the uncertainties of sTIACs ($U_{sTIAC})$and rTIAC ($U_{rTIAC})$ values (Eq. 5). The uncertainty of RMSE ($U_{RMSE})$ is derived through error propagation from both the mean and standard deviation of the RD values (Eq. 6), where index ($i,j$) is the $i$-th patient and the j-th time point. Meanwhile, the uncertainty of MAPE ($U_{MAPE})$ is computed cumulatively for each pair of estimated and reference data points (Eq. 7) and the standard deviation of RD values (${SD}_{RD}$).

**Part B. Fitting**

To determine the minimum error location over time, three polynomial models with increasing complexity were applied:

$y_{1}(x)=a_{1}+c_{1}{(x-b_{1})}^{2}$	(S6)

$y_{2}(x)= a_{2}+c_{2}{(x-b_{2})}^{2}+d_{2}{(x-b_{2})}^{4}$	(S7)

$y_{3}(x)= a_{3}+c_{3}{(x-b_{3})}^{2}+d_{3}{(x-b_{3})}^{4}+e_{3}{(x-b_{3})}^{6}$	(S8)

In these equations, parameter b represents the minimum location of the RMSE-time and MAPE-time curves and was interpreted as the estimated optimal imaging time. The standard error of b was derived from the parameter covariance matrix obtained in SAAM II.

To support the model selection process discussed in the main text, we describe the fitting approach and the criteria used to evaluate model performance. Furthermore, to select the best models (S6), (S7), and (S8) to describe the MAPE and RMSE data, the Coefficient of Variation (CV) criterion is used. The CV for each fitted parameter was automatically computed by SAAM II [3,4]. The CV value obtained for each parameter must be less than 50%. A CV value of 𝐶𝑉 < 25% is considered precise, and 𝐶𝑉 < 50% acceptable.

**Part C. Supplemental Tables**

**Table S1.** Model parameters estimated from by fitting the time-activity data with the best-fit function (the SOEF model, Eq. 1 of the main text) adopted from [5], used to generate virtual patients.

| **Model parameters (unit)** | **Fixed effect (%CV)** | **Random effect  (variance, inter-patient variability)** |
| --- | --- | --- |
| $A_{1}$ | 1.64 (29.8) | 0.22 |
| $A_{2}$ | 0.84 (27.2) | 0.16 |
| $A_{3}$ | 0.49 (43.6) | 0.38 |
| $\lambda_{1}$ | 6.03 x 10^-2^ (31.3) | 0.30 |
| $\lambda_{2}$ | 9.07 x 10^-3^ (22.6) | 0.08 |
| $\lambda_{3}$ | 7.19 x 10^-2^ (42.5) | 0.52 |
| Residual variability | 0 | 0.079 |

**Table S2.** Model selection results based on RMSE and MAPE, with the maximum coefficient of variation (CV) for each model.

| **Models** | **RMSE** | **MAPE** |
| --- | --- | --- |
|  | **CV Maximum (%)** | **CV Maximum (%)** |
| S6 | 7.20 | 7.42 |
| S7 | 256 | 520 |
| S8 | 263 | 165 |

**Table S3.** Polynomial fitting parameters (a–c) obtained from MAPE- and RMSE-based curve fitting for polynomial models (S6).

| **Metric** | **MAPE** | | |
| --- | --- | --- | --- |
|  | **a ± SE(a)** | **b ± SE(b)** | **c ± SE(c)** |
| S6 | 9.30 ± 0.09 | 50.04 ± 0.69 | 0.0025 ± 0.0002 |
| **Metric** | **RMSE** | | |
|  | **a ± SE(a)** | **b ± SE(b)** | **c ± SE(c)** |
| S6 | 11.71 ± 0.14 | 49.67 ± 0.68 | 0.0031 ± 0.0002 |

**Tables S4.** Virtual patient parameters generated using the parameters in Table S1

| **Virtual Patient** | $A_{1}$ | $A_{2}$ | $A_{3}$ | $\lambda_{1}$ | $\lambda_{2}$ | $\lambda_{3}$ |
| --- | --- | --- | --- | --- | --- | --- |
| 1 | 0.0162 | 0.0056 | 0.0042 | 0.0670 | 0.0101 | 0.0908 |
| 2 | 0.0163 | 0.0061 | 0.0040 | 0.0611 | 0.0098 | 0.0813 |
| 3 | 0.0173 | 0.0065 | 0.0047 | 0.0592 | 0.0095 | 0.0828 |
| 4 | 0.0179 | 0.0088 | 0.0040 | 0.0469 | 0.0089 | 0.0912 |
| 5 | 0.0176 | 0.0083 | 0.0041 | 0.0494 | 0.0090 | 0.0906 |
| 6 | 0.0168 | 0.0069 | 0.0042 | 0.0563 | 0.0095 | 0.0813 |
| 7 | 0.0252 | 0.0127 | 0.0052 | 0.0316 | 0.0082 | 0.0811 |
| 8 | 0.0217 | 0.0113 | 0.0039 | 0.0337 | 0.0084 | 0.0934 |
| 9 | 0.0188 | 0.0096 | 0.0041 | 0.0425 | 0.0087 | 0.0848 |
| 10 | 0.0173 | 0.0071 | 0.0045 | 0.0562 | 0.0093 | 0.0844 |
| 11 | 0.0228 | 0.0120 | 0.0044 | 0.0338 | 0.0083 | 0.0851 |
| 12 | 0.0177 | 0.0085 | 0.0043 | 0.0491 | 0.0090 | 0.0946 |
| 13 | 0.0189 | 0.0094 | 0.0039 | 0.0424 | 0.0087 | 0.0848 |
| 14 | 0.0179 | 0.0088 | 0.0041 | 0.0468 | 0.0089 | 0.0905 |
| 15 | 0.0179 | 0.0089 | 0.0042 | 0.0469 | 0.0089 | 0.0945 |
| 16 | 0.0213 | 0.0113 | 0.0042 | 0.0357 | 0.0084 | 0.0894 |
| 17 | 0.0166 | 0.0066 | 0.0041 | 0.0585 | 0.0096 | 0.0844 |
| 18 | 0.0195 | 0.0102 | 0.0042 | 0.0407 | 0.0086 | 0.0891 |
| 19 | 0.0169 | 0.0072 | 0.0043 | 0.0553 | 0.0094 | 0.0864 |
| 20 | 0.0166 | 0.0064 | 0.0043 | 0.0607 | 0.0097 | 0.0855 |
| 21 | 0.0216 | 0.0113 | 0.0047 | 0.0365 | 0.0084 | 0.0838 |
| 22 | 0.0219 | 0.0112 | 0.0050 | 0.0369 | 0.0084 | 0.0806 |
| 23 | 0.0196 | 0.0101 | 0.0043 | 0.0405 | 0.0086 | 0.0868 |
| 24 | 0.0180 | 0.0082 | 0.0044 | 0.0498 | 0.0090 | 0.0845 |
| 25 | 0.0220 | 0.0116 | 0.0041 | 0.0337 | 0.0084 | 0.0920 |
| 26 | 0.0169 | 0.0077 | 0.0040 | 0.0525 | 0.0092 | 0.0912 |
| 27 | 0.0169 | 0.0072 | 0.0042 | 0.0552 | 0.0094 | 0.0865 |
| 28 | 0.0165 | 0.0067 | 0.0040 | 0.0571 | 0.0095 | 0.0837 |
| 29 | 0.0170 | 0.0073 | 0.0043 | 0.0552 | 0.0094 | 0.0884 |
| 30 | 0.0209 | 0.0111 | 0.0044 | 0.0372 | 0.0085 | 0.0871 |
| 31 | 0.0187 | 0.0095 | 0.0040 | 0.0433 | 0.0087 | 0.0870 |
| 32 | 0.0171 | 0.0065 | 0.0046 | 0.0592 | 0.0095 | 0.0831 |
| 33 | 0.0169 | 0.0077 | 0.0041 | 0.0531 | 0.0092 | 0.0932 |
| 34 | 0.0194 | 0.0104 | 0.0042 | 0.0408 | 0.0087 | 0.0970 |
| 35 | 0.0165 | 0.0065 | 0.0042 | 0.0597 | 0.0096 | 0.0863 |
| 36 | 0.0173 | 0.0080 | 0.0042 | 0.0516 | 0.0092 | 0.0906 |
| 37 | 0.0198 | 0.0105 | 0.0040 | 0.0387 | 0.0085 | 0.0915 |
| 38 | 0.0169 | 0.0077 | 0.0040 | 0.0531 | 0.0093 | 0.0934 |
| 39 | 0.0172 | 0.0075 | 0.0044 | 0.0545 | 0.0094 | 0.0857 |
| 40 | 0.0167 | 0.0075 | 0.0041 | 0.0549 | 0.0094 | 0.0963 |
| 41 | 0.0367 | 0.0128 | 0.0045 | 0.0228 | 0.0082 | 0.0832 |
| 42 | 0.0176 | 0.0084 | 0.0038 | 0.0482 | 0.0089 | 0.0900 |
| 43 | 0.0167 | 0.0071 | 0.0040 | 0.0556 | 0.0094 | 0.0857 |
| 44 | 0.0165 | 0.0073 | 0.0039 | 0.0550 | 0.0094 | 0.0943 |
| 45 | 0.0205 | 0.0111 | 0.0040 | 0.0366 | 0.0085 | 0.0944 |
| 46 | 0.0239 | 0.0120 | 0.0041 | 0.0309 | 0.0084 | 0.0927 |
| 47 | 0.0190 | 0.0098 | 0.0043 | 0.0430 | 0.0087 | 0.0883 |
| 48 | 0.0159 | 0.0048 | 0.0045 | 0.0725 | 0.0107 | 0.0785 |
| 49 | 0.0183 | 0.0092 | 0.0041 | 0.0449 | 0.0088 | 0.0896 |
| 50 | 0.0176 | 0.0083 | 0.0041 | 0.0486 | 0.0090 | 0.0843 |
| 51 | 0.0185 | 0.0093 | 0.0041 | 0.0441 | 0.0088 | 0.0865 |
| 52 | 0.0179 | 0.0087 | 0.0043 | 0.0477 | 0.0090 | 0.0892 |
| 53 | 0.0183 | 0.0093 | 0.0042 | 0.0454 | 0.0089 | 0.0915 |
| 54 | 0.0175 | 0.0081 | 0.0041 | 0.0508 | 0.0091 | 0.0897 |
| 55 | 0.0188 | 0.0094 | 0.0043 | 0.0440 | 0.0088 | 0.0862 |
| 56 | 0.0168 | 0.0072 | 0.0040 | 0.0554 | 0.0093 | 0.0880 |
| 57 | 0.0230 | 0.0115 | 0.0042 | 0.0323 | 0.0083 | 0.0855 |
| 58 | 0.0174 | 0.0082 | 0.0041 | 0.0503 | 0.0091 | 0.0909 |
| 59 | 0.0193 | 0.0104 | 0.0040 | 0.0403 | 0.0087 | 0.0933 |
| 60 | 0.0220 | 0.0118 | 0.0041 | 0.0338 | 0.0084 | 0.0982 |
| 61 | 0.0168 | 0.0063 | 0.0044 | 0.0608 | 0.0097 | 0.0836 |
| 62 | 0.0165 | 0.0068 | 0.0041 | 0.0589 | 0.0096 | 0.0948 |
| 63 | 0.0180 | 0.0091 | 0.0039 | 0.0450 | 0.0088 | 0.0936 |
| 64 | 0.0184 | 0.0076 | 0.0051 | 0.0524 | 0.0091 | 0.0765 |
| 65 | 0.0173 | 0.0080 | 0.0040 | 0.0507 | 0.0091 | 0.0877 |
| 66 | 0.0213 | 0.0112 | 0.0043 | 0.0360 | 0.0084 | 0.0886 |
| 67 | 0.0177 | 0.0084 | 0.0041 | 0.0483 | 0.0090 | 0.0849 |
| 68 | 0.0177 | 0.0084 | 0.0042 | 0.0487 | 0.0091 | 0.0839 |
| 69 | 0.0167 | 0.0072 | 0.0040 | 0.0556 | 0.0094 | 0.0891 |
| 70 | 0.0213 | 0.0114 | 0.0041 | 0.0354 | 0.0084 | 0.0943 |
| 71 | 0.0180 | 0.0086 | 0.0041 | 0.0476 | 0.0089 | 0.0885 |
| 72 | 0.0302 | 0.0130 | 0.0042 | 0.0254 | 0.0083 | 0.0965 |
| 73 | 0.0168 | 0.0071 | 0.0041 | 0.0562 | 0.0094 | 0.0902 |
| 74 | 0.0167 | 0.0066 | 0.0042 | 0.0585 | 0.0096 | 0.0831 |
| 75 | 0.0169 | 0.0077 | 0.0040 | 0.0534 | 0.0093 | 0.0944 |
| 76 | 0.0170 | 0.0076 | 0.0038 | 0.0527 | 0.0092 | 0.0899 |
| 77 | 0.0172 | 0.0079 | 0.0041 | 0.0516 | 0.0092 | 0.0907 |
| 78 | 0.0169 | 0.0076 | 0.0039 | 0.0529 | 0.0092 | 0.0901 |
| 79 | 0.0180 | 0.0082 | 0.0047 | 0.0512 | 0.0091 | 0.0873 |
| 80 | 0.0179 | 0.0086 | 0.0043 | 0.0481 | 0.0090 | 0.0881 |
| 81 | 0.0179 | 0.0088 | 0.0039 | 0.0463 | 0.0089 | 0.0889 |
| 82 | 0.0272 | 0.0126 | 0.0042 | 0.0276 | 0.0083 | 0.0922 |
| 83 | 0.0175 | 0.0083 | 0.0041 | 0.0497 | 0.0090 | 0.0921 |
| 84 | 0.0159 | 0.0048 | 0.0042 | 0.0714 | 0.0105 | 0.0837 |
| 85 | 0.0170 | 0.0079 | 0.0039 | 0.0513 | 0.0091 | 0.0917 |
| 86 | 0.0176 | 0.0080 | 0.0044 | 0.0513 | 0.0092 | 0.0895 |
| 87 | 0.0184 | 0.0093 | 0.0042 | 0.0451 | 0.0088 | 0.0935 |
| 88 | 0.0172 | 0.0080 | 0.0041 | 0.0512 | 0.0092 | 0.0864 |
| 89 | 0.0158 | 0.0057 | 0.0040 | 0.0658 | 0.0101 | 0.0917 |
| 90 | 0.0218 | 0.0116 | 0.0041 | 0.0343 | 0.0084 | 0.0892 |
| 91 | 0.0178 | 0.0087 | 0.0044 | 0.0484 | 0.0090 | 0.0962 |
| 92 | 0.0231 | 0.0119 | 0.0040 | 0.0318 | 0.0083 | 0.0957 |
| 93 | 0.0176 | 0.0081 | 0.0044 | 0.0510 | 0.0091 | 0.0886 |
| 94 | 0.0208 | 0.0110 | 0.0041 | 0.0366 | 0.0085 | 0.0924 |
| 95 | 0.0162 | 0.0061 | 0.0041 | 0.0629 | 0.0099 | 0.0951 |
| 96 | 0.0195 | 0.0104 | 0.0039 | 0.0393 | 0.0086 | 0.0940 |
| 97 | 0.0161 | 0.0056 | 0.0042 | 0.0663 | 0.0101 | 0.0894 |
| 98 | 0.0174 | 0.0078 | 0.0042 | 0.0521 | 0.0092 | 0.0887 |
| 99 | 0.0180 | 0.0083 | 0.0044 | 0.0489 | 0.0090 | 0.0838 |
| 100 | 0.0168 | 0.0071 | 0.0043 | 0.0569 | 0.0095 | 0.0921 |
| 101 | 0.0157 | 0.0054 | 0.0038 | 0.0672 | 0.0102 | 0.0902 |
| 102 | 0.0225 | 0.0117 | 0.0043 | 0.0339 | 0.0084 | 0.0823 |
| 103 | 0.0210 | 0.0114 | 0.0042 | 0.0363 | 0.0085 | 0.0978 |
| 104 | 0.0176 | 0.0081 | 0.0042 | 0.0506 | 0.0090 | 0.0879 |
| 105 | 0.0193 | 0.0101 | 0.0039 | 0.0398 | 0.0086 | 0.0928 |
| 106 | 0.0171 | 0.0074 | 0.0042 | 0.0544 | 0.0093 | 0.0871 |
| 107 | 0.0166 | 0.0070 | 0.0039 | 0.0557 | 0.0094 | 0.0851 |
| 108 | 0.0237 | 0.0121 | 0.0043 | 0.0315 | 0.0083 | 0.0897 |
| 109 | 0.0223 | 0.0116 | 0.0041 | 0.0338 | 0.0083 | 0.0876 |
| 110 | 0.0176 | 0.0079 | 0.0043 | 0.0513 | 0.0091 | 0.0844 |
| 111 | 0.0167 | 0.0061 | 0.0044 | 0.0614 | 0.0097 | 0.0824 |
| 112 | 0.0169 | 0.0075 | 0.0043 | 0.0545 | 0.0094 | 0.0886 |
| 113 | 0.0182 | 0.0089 | 0.0041 | 0.0455 | 0.0088 | 0.0869 |
| 114 | 0.0192 | 0.0100 | 0.0041 | 0.0407 | 0.0087 | 0.0866 |
| 115 | 0.0183 | 0.0092 | 0.0041 | 0.0451 | 0.0088 | 0.0897 |
| 116 | 0.0206 | 0.0111 | 0.0041 | 0.0369 | 0.0085 | 0.0969 |
| 117 | 0.0167 | 0.0066 | 0.0043 | 0.0585 | 0.0096 | 0.0848 |
| 118 | 0.0172 | 0.0075 | 0.0043 | 0.0536 | 0.0093 | 0.0868 |
| 119 | 0.0203 | 0.0107 | 0.0041 | 0.0380 | 0.0085 | 0.0873 |
| 120 | 0.0189 | 0.0096 | 0.0043 | 0.0432 | 0.0087 | 0.0904 |
| 121 | 0.0332 | 0.0128 | 0.0042 | 0.0241 | 0.0083 | 0.0899 |
| 122 | 0.0304 | 0.0125 | 0.0042 | 0.0254 | 0.0082 | 0.0864 |
| 123 | 0.0168 | 0.0072 | 0.0042 | 0.0563 | 0.0095 | 0.0897 |
| 124 | 0.0184 | 0.0095 | 0.0042 | 0.0439 | 0.0088 | 0.0945 |
| 125 | 0.0179 | 0.0083 | 0.0044 | 0.0496 | 0.0090 | 0.0869 |
| 126 | 0.0174 | 0.0072 | 0.0047 | 0.0555 | 0.0093 | 0.0848 |
| 127 | 0.0189 | 0.0093 | 0.0045 | 0.0449 | 0.0088 | 0.0841 |
| 128 | 0.0179 | 0.0078 | 0.0048 | 0.0526 | 0.0092 | 0.0838 |
| 129 | 0.0166 | 0.0066 | 0.0042 | 0.0594 | 0.0096 | 0.0865 |
| 130 | 0.0178 | 0.0085 | 0.0041 | 0.0482 | 0.0090 | 0.0893 |
| 131 | 0.0163 | 0.0060 | 0.0044 | 0.0629 | 0.0100 | 0.0857 |
| 132 | 0.0180 | 0.0090 | 0.0041 | 0.0464 | 0.0089 | 0.0898 |
| 133 | 0.0177 | 0.0082 | 0.0044 | 0.0510 | 0.0091 | 0.0876 |
| 134 | 0.0230 | 0.0119 | 0.0041 | 0.0323 | 0.0083 | 0.0890 |
| 135 | 0.0161 | 0.0063 | 0.0040 | 0.0614 | 0.0098 | 0.0923 |
| 136 | 0.0208 | 0.0108 | 0.0043 | 0.0377 | 0.0085 | 0.0848 |
| 137 | 0.0189 | 0.0090 | 0.0047 | 0.0461 | 0.0088 | 0.0845 |
| 138 | 0.0188 | 0.0096 | 0.0043 | 0.0434 | 0.0088 | 0.0908 |
| 139 | 0.0167 | 0.0067 | 0.0042 | 0.0580 | 0.0095 | 0.0871 |
| 140 | 0.0161 | 0.0053 | 0.0043 | 0.0676 | 0.0102 | 0.0806 |
| 141 | 0.0187 | 0.0096 | 0.0040 | 0.0429 | 0.0087 | 0.0915 |
| 142 | 0.0323 | 0.0128 | 0.0043 | 0.0244 | 0.0082 | 0.0899 |
| 143 | 0.0178 | 0.0087 | 0.0041 | 0.0475 | 0.0090 | 0.0884 |
| 144 | 0.0169 | 0.0076 | 0.0042 | 0.0538 | 0.0094 | 0.0932 |
| 145 | 0.0164 | 0.0058 | 0.0043 | 0.0642 | 0.0099 | 0.0843 |
| 146 | 0.0240 | 0.0123 | 0.0044 | 0.0315 | 0.0083 | 0.0888 |
| 147 | 0.0225 | 0.0118 | 0.0043 | 0.0336 | 0.0084 | 0.0889 |
| 148 | 0.0220 | 0.0115 | 0.0043 | 0.0347 | 0.0084 | 0.0869 |
| 149 | 0.0171 | 0.0074 | 0.0043 | 0.0555 | 0.0094 | 0.0910 |
| 150 | 0.0174 | 0.0083 | 0.0042 | 0.0500 | 0.0091 | 0.0929 |
| 151 | 0.0252 | 0.0124 | 0.0046 | 0.0305 | 0.0082 | 0.0855 |
| 152 | 0.0183 | 0.0090 | 0.0043 | 0.0466 | 0.0089 | 0.0905 |
| 153 | 0.0286 | 0.0132 | 0.0044 | 0.0271 | 0.0082 | 0.0894 |
| 154 | 0.0169 | 0.0073 | 0.0043 | 0.0549 | 0.0094 | 0.0898 |
| 155 | 0.0207 | 0.0110 | 0.0039 | 0.0362 | 0.0085 | 0.0875 |
| 156 | 0.0176 | 0.0082 | 0.0042 | 0.0499 | 0.0091 | 0.0866 |
| 157 | 0.0170 | 0.0078 | 0.0041 | 0.0522 | 0.0093 | 0.0917 |
| 158 | 0.0184 | 0.0090 | 0.0043 | 0.0465 | 0.0088 | 0.0888 |
| 159 | 0.0175 | 0.0068 | 0.0047 | 0.0579 | 0.0095 | 0.0832 |
| 160 | 0.0202 | 0.0107 | 0.0040 | 0.0379 | 0.0085 | 0.0874 |
| 161 | 0.0203 | 0.0105 | 0.0043 | 0.0388 | 0.0085 | 0.0856 |
| 162 | 0.0156 | 0.0049 | 0.0041 | 0.0716 | 0.0106 | 0.0879 |
| 163 | 0.0175 | 0.0083 | 0.0040 | 0.0489 | 0.0090 | 0.0891 |
| 164 | 0.0175 | 0.0084 | 0.0038 | 0.0480 | 0.0090 | 0.0881 |
| 165 | 0.0208 | 0.0111 | 0.0041 | 0.0365 | 0.0085 | 0.0914 |
| 166 | 0.0168 | 0.0060 | 0.0047 | 0.0634 | 0.0099 | 0.0840 |
| 167 | 0.0162 | 0.0054 | 0.0043 | 0.0682 | 0.0102 | 0.0901 |
| 168 | 0.0172 | 0.0076 | 0.0045 | 0.0546 | 0.0094 | 0.0942 |
| 169 | 0.0186 | 0.0094 | 0.0043 | 0.0455 | 0.0088 | 0.0931 |
| 170 | 0.0168 | 0.0079 | 0.0038 | 0.0512 | 0.0092 | 0.0975 |
| 171 | 0.0192 | 0.0102 | 0.0040 | 0.0409 | 0.0087 | 0.0943 |
| 172 | 0.0164 | 0.0066 | 0.0040 | 0.0593 | 0.0096 | 0.0881 |
| 173 | 0.0217 | 0.0113 | 0.0042 | 0.0348 | 0.0084 | 0.0846 |
| 174 | 0.0162 | 0.0064 | 0.0039 | 0.0602 | 0.0097 | 0.0891 |
| 175 | 0.0169 | 0.0077 | 0.0038 | 0.0522 | 0.0092 | 0.0921 |
| 176 | 0.0168 | 0.0074 | 0.0040 | 0.0543 | 0.0093 | 0.0915 |
| 177 | 0.0166 | 0.0073 | 0.0040 | 0.0561 | 0.0094 | 0.0995 |
| 178 | 0.0225 | 0.0118 | 0.0041 | 0.0333 | 0.0083 | 0.0866 |
| 179 | 0.0174 | 0.0081 | 0.0039 | 0.0493 | 0.0090 | 0.0875 |
| 180 | 0.0187 | 0.0096 | 0.0041 | 0.0430 | 0.0087 | 0.0894 |
| 181 | 0.0186 | 0.0095 | 0.0040 | 0.0434 | 0.0088 | 0.0920 |
| 182 | 0.0185 | 0.0097 | 0.0038 | 0.0427 | 0.0088 | 0.0917 |
| 183 | 0.0207 | 0.0113 | 0.0039 | 0.0361 | 0.0085 | 0.0994 |
| 184 | 0.0179 | 0.0087 | 0.0042 | 0.0478 | 0.0089 | 0.0926 |
| 185 | 0.0179 | 0.0089 | 0.0041 | 0.0466 | 0.0089 | 0.0927 |
| 186 | 0.0173 | 0.0074 | 0.0044 | 0.0547 | 0.0093 | 0.0874 |
| 187 | 0.0180 | 0.0086 | 0.0042 | 0.0480 | 0.0089 | 0.0886 |
| 188 | 0.0232 | 0.0120 | 0.0047 | 0.0335 | 0.0083 | 0.0864 |
| 189 | 0.0172 | 0.0079 | 0.0039 | 0.0513 | 0.0091 | 0.0866 |
| 190 | 0.0170 | 0.0075 | 0.0041 | 0.0546 | 0.0093 | 0.0909 |
| 191 | 0.0172 | 0.0078 | 0.0042 | 0.0527 | 0.0092 | 0.0929 |
| 192 | 0.0248 | 0.0124 | 0.0041 | 0.0301 | 0.0083 | 0.0885 |
| 193 | 0.0165 | 0.0067 | 0.0041 | 0.0584 | 0.0096 | 0.0886 |
| 194 | 0.0187 | 0.0096 | 0.0041 | 0.0429 | 0.0088 | 0.0909 |
| 195 | 0.0165 | 0.0061 | 0.0042 | 0.0621 | 0.0098 | 0.0879 |
| 196 | 0.0171 | 0.0071 | 0.0045 | 0.0564 | 0.0094 | 0.0870 |
| 197 | 0.0212 | 0.0111 | 0.0040 | 0.0356 | 0.0084 | 0.0861 |
| 198 | 0.0166 | 0.0068 | 0.0042 | 0.0588 | 0.0095 | 0.0925 |
| 199 | 0.0166 | 0.0070 | 0.0040 | 0.0572 | 0.0095 | 0.0905 |
| 200 | 0.0384 | 0.0123 | 0.0042 | 0.0220 | 0.0083 | 0.0882 |
| 201 | 0.0194 | 0.0102 | 0.0040 | 0.0404 | 0.0086 | 0.0925 |
| 202 | 0.0162 | 0.0060 | 0.0042 | 0.0638 | 0.0099 | 0.0882 |
| 203 | 0.0169 | 0.0073 | 0.0042 | 0.0554 | 0.0094 | 0.0900 |
| 204 | 0.0193 | 0.0100 | 0.0042 | 0.0411 | 0.0086 | 0.0880 |
| 205 | 0.0172 | 0.0080 | 0.0040 | 0.0514 | 0.0091 | 0.0924 |
| 206 | 0.0191 | 0.0098 | 0.0042 | 0.0422 | 0.0087 | 0.0915 |
| 207 | 0.0251 | 0.0122 | 0.0037 | 0.0290 | 0.0083 | 0.0958 |
| 208 | 0.0191 | 0.0100 | 0.0042 | 0.0419 | 0.0087 | 0.0912 |
| 209 | 0.0157 | 0.0052 | 0.0040 | 0.0697 | 0.0104 | 0.0909 |
| 210 | 0.0181 | 0.0076 | 0.0050 | 0.0530 | 0.0092 | 0.0787 |
| 211 | 0.0186 | 0.0093 | 0.0044 | 0.0450 | 0.0088 | 0.0918 |
| 212 | 0.0291 | 0.0124 | 0.0041 | 0.0259 | 0.0082 | 0.0893 |
| 213 | 0.0166 | 0.0070 | 0.0040 | 0.0577 | 0.0095 | 0.0947 |
| 214 | 0.0286 | 0.0123 | 0.0040 | 0.0262 | 0.0083 | 0.0905 |
| 215 | 0.0165 | 0.0064 | 0.0042 | 0.0604 | 0.0097 | 0.0894 |
| 216 | 0.0186 | 0.0094 | 0.0041 | 0.0439 | 0.0088 | 0.0858 |
| 217 | 0.0227 | 0.0115 | 0.0042 | 0.0330 | 0.0083 | 0.0849 |
| 218 | 0.0164 | 0.0067 | 0.0039 | 0.0587 | 0.0096 | 0.0890 |
| 219 | 0.0174 | 0.0084 | 0.0040 | 0.0486 | 0.0090 | 0.0913 |
| 220 | 0.0164 | 0.0061 | 0.0043 | 0.0626 | 0.0098 | 0.0879 |
| 221 | 0.0174 | 0.0081 | 0.0040 | 0.0504 | 0.0091 | 0.0925 |
| 222 | 0.0173 | 0.0082 | 0.0039 | 0.0495 | 0.0091 | 0.0911 |
| 223 | 0.0172 | 0.0073 | 0.0044 | 0.0546 | 0.0093 | 0.0850 |
| 224 | 0.0197 | 0.0104 | 0.0042 | 0.0399 | 0.0086 | 0.0889 |
| 225 | 0.0253 | 0.0125 | 0.0046 | 0.0301 | 0.0082 | 0.0849 |
| 226 | 0.0167 | 0.0057 | 0.0046 | 0.0641 | 0.0099 | 0.0798 |
| 227 | 0.0160 | 0.0055 | 0.0042 | 0.0663 | 0.0101 | 0.0851 |
| 228 | 0.0300 | 0.0129 | 0.0041 | 0.0252 | 0.0082 | 0.0880 |
| 229 | 0.0167 | 0.0065 | 0.0045 | 0.0603 | 0.0097 | 0.0886 |
| 230 | 0.0232 | 0.0121 | 0.0040 | 0.0318 | 0.0083 | 0.0879 |
| 231 | 0.0169 | 0.0066 | 0.0045 | 0.0596 | 0.0096 | 0.0854 |
| 232 | 0.0183 | 0.0088 | 0.0045 | 0.0480 | 0.0090 | 0.0895 |
| 233 | 0.0177 | 0.0083 | 0.0043 | 0.0490 | 0.0090 | 0.0888 |
| 234 | 0.0170 | 0.0072 | 0.0043 | 0.0549 | 0.0094 | 0.0830 |
| 235 | 0.0177 | 0.0087 | 0.0040 | 0.0474 | 0.0090 | 0.0911 |
| 236 | 0.0174 | 0.0081 | 0.0042 | 0.0509 | 0.0091 | 0.0957 |
| 237 | 0.0161 | 0.0062 | 0.0039 | 0.0605 | 0.0098 | 0.0823 |
| 238 | 0.0191 | 0.0097 | 0.0041 | 0.0420 | 0.0087 | 0.0843 |
| 239 | 0.0183 | 0.0089 | 0.0042 | 0.0460 | 0.0088 | 0.0883 |
| 240 | 0.0169 | 0.0077 | 0.0039 | 0.0529 | 0.0092 | 0.0935 |
| 241 | 0.0165 | 0.0061 | 0.0043 | 0.0611 | 0.0097 | 0.0832 |
| 242 | 0.0195 | 0.0100 | 0.0042 | 0.0408 | 0.0086 | 0.0876 |
| 243 | 0.0221 | 0.0116 | 0.0043 | 0.0343 | 0.0084 | 0.0861 |
| 244 | 0.0162 | 0.0054 | 0.0043 | 0.0667 | 0.0101 | 0.0837 |
| 245 | 0.0221 | 0.0116 | 0.0041 | 0.0341 | 0.0084 | 0.0892 |
| 246 | 0.0173 | 0.0083 | 0.0040 | 0.0502 | 0.0091 | 0.0943 |
| 247 | 0.0191 | 0.0095 | 0.0046 | 0.0441 | 0.0087 | 0.0841 |
| 248 | 0.0172 | 0.0078 | 0.0041 | 0.0515 | 0.0092 | 0.0853 |
| 249 | 0.0341 | 0.0127 | 0.0043 | 0.0237 | 0.0082 | 0.0863 |
| 250 | 0.0180 | 0.0086 | 0.0042 | 0.0475 | 0.0089 | 0.0879 |
| 251 | 0.0171 | 0.0077 | 0.0041 | 0.0527 | 0.0092 | 0.0900 |
| 252 | 0.0168 | 0.0072 | 0.0042 | 0.0561 | 0.0095 | 0.0897 |
| 253 | 0.0178 | 0.0086 | 0.0041 | 0.0484 | 0.0090 | 0.0910 |
| 254 | 0.0196 | 0.0106 | 0.0043 | 0.0398 | 0.0087 | 0.0976 |
| 255 | 0.0194 | 0.0102 | 0.0041 | 0.0403 | 0.0086 | 0.0936 |
| 256 | 0.0190 | 0.0099 | 0.0041 | 0.0424 | 0.0087 | 0.0880 |
| 257 | 0.0191 | 0.0096 | 0.0044 | 0.0432 | 0.0087 | 0.0844 |
| 258 | 0.0167 | 0.0073 | 0.0038 | 0.0543 | 0.0093 | 0.0867 |
| 259 | 0.0162 | 0.0068 | 0.0037 | 0.0582 | 0.0096 | 0.0959 |
| 260 | 0.0180 | 0.0087 | 0.0044 | 0.0483 | 0.0090 | 0.0901 |
| 261 | 0.0196 | 0.0104 | 0.0041 | 0.0407 | 0.0086 | 0.0888 |
| 262 | 0.0160 | 0.0054 | 0.0041 | 0.0679 | 0.0102 | 0.0896 |
| 263 | 0.0170 | 0.0075 | 0.0041 | 0.0541 | 0.0093 | 0.0899 |
| 264 | 0.0175 | 0.0085 | 0.0040 | 0.0483 | 0.0090 | 0.0915 |
| 265 | 0.0159 | 0.0048 | 0.0042 | 0.0727 | 0.0106 | 0.0898 |
| 266 | 0.0228 | 0.0119 | 0.0045 | 0.0338 | 0.0084 | 0.0843 |
| 267 | 0.0169 | 0.0073 | 0.0042 | 0.0557 | 0.0094 | 0.0891 |
| 268 | 0.0168 | 0.0071 | 0.0041 | 0.0560 | 0.0094 | 0.0880 |
| 269 | 0.0208 | 0.0111 | 0.0040 | 0.0363 | 0.0085 | 0.0949 |
| 270 | 0.0166 | 0.0070 | 0.0040 | 0.0569 | 0.0095 | 0.0885 |
| 271 | 0.0204 | 0.0108 | 0.0043 | 0.0380 | 0.0086 | 0.0900 |
| 272 | 0.0176 | 0.0085 | 0.0041 | 0.0492 | 0.0091 | 0.0928 |
| 273 | 0.0204 | 0.0109 | 0.0042 | 0.0378 | 0.0085 | 0.0912 |
| 274 | 0.0230 | 0.0118 | 0.0039 | 0.0316 | 0.0084 | 0.0940 |
| 275 | 0.0176 | 0.0080 | 0.0043 | 0.0503 | 0.0091 | 0.0843 |
| 276 | 0.0164 | 0.0062 | 0.0043 | 0.0614 | 0.0098 | 0.0853 |
| 277 | 0.0178 | 0.0083 | 0.0044 | 0.0497 | 0.0090 | 0.0882 |
| 278 | 0.0164 | 0.0069 | 0.0040 | 0.0575 | 0.0096 | 0.0901 |
| 279 | 0.0200 | 0.0106 | 0.0042 | 0.0388 | 0.0086 | 0.0914 |
| 280 | 0.0168 | 0.0066 | 0.0045 | 0.0600 | 0.0097 | 0.0882 |
| 281 | 0.0169 | 0.0071 | 0.0042 | 0.0561 | 0.0095 | 0.0848 |
| 282 | 0.0179 | 0.0087 | 0.0040 | 0.0470 | 0.0089 | 0.0900 |
| 283 | 0.0169 | 0.0071 | 0.0043 | 0.0564 | 0.0094 | 0.0855 |
| 284 | 0.0157 | 0.0048 | 0.0042 | 0.0731 | 0.0106 | 0.0929 |
| 285 | 0.0184 | 0.0089 | 0.0044 | 0.0462 | 0.0089 | 0.0832 |
| 286 | 0.0241 | 0.0123 | 0.0042 | 0.0308 | 0.0083 | 0.0981 |
| 287 | 0.0194 | 0.0100 | 0.0041 | 0.0410 | 0.0086 | 0.0851 |
| 288 | 0.0176 | 0.0081 | 0.0043 | 0.0511 | 0.0091 | 0.0909 |
| 289 | 0.0196 | 0.0103 | 0.0041 | 0.0406 | 0.0086 | 0.0906 |
| 290 | 0.0173 | 0.0072 | 0.0043 | 0.0548 | 0.0093 | 0.0832 |
| 291 | 0.0175 | 0.0082 | 0.0041 | 0.0496 | 0.0091 | 0.0872 |
| 292 | 0.0196 | 0.0102 | 0.0042 | 0.0406 | 0.0086 | 0.0895 |
| 293 | 0.0170 | 0.0068 | 0.0044 | 0.0582 | 0.0095 | 0.0857 |
| 294 | 0.0167 | 0.0070 | 0.0042 | 0.0574 | 0.0095 | 0.0947 |
| 295 | 0.0173 | 0.0078 | 0.0040 | 0.0518 | 0.0091 | 0.0897 |
| 296 | 0.0174 | 0.0078 | 0.0043 | 0.0513 | 0.0092 | 0.0821 |
| 297 | 0.0186 | 0.0095 | 0.0041 | 0.0436 | 0.0088 | 0.0931 |
| 298 | 0.0167 | 0.0071 | 0.0040 | 0.0560 | 0.0094 | 0.0906 |
| 299 | 0.0248 | 0.0121 | 0.0040 | 0.0296 | 0.0083 | 0.0919 |
| 300 | 0.0174 | 0.0082 | 0.0041 | 0.0502 | 0.0091 | 0.0883 |
| 301 | 0.0175 | 0.0079 | 0.0043 | 0.0517 | 0.0092 | 0.0856 |
| 302 | 0.0207 | 0.0110 | 0.0041 | 0.0369 | 0.0085 | 0.0900 |
| 303 | 0.0158 | 0.0047 | 0.0041 | 0.0732 | 0.0106 | 0.0868 |
| 304 | 0.0172 | 0.0079 | 0.0040 | 0.0516 | 0.0091 | 0.0917 |
| 305 | 0.0173 | 0.0074 | 0.0042 | 0.0540 | 0.0092 | 0.0846 |
| 306 | 0.0192 | 0.0101 | 0.0042 | 0.0416 | 0.0087 | 0.0922 |
| 307 | 0.0192 | 0.0102 | 0.0037 | 0.0395 | 0.0086 | 0.0949 |
| 308 | 0.0167 | 0.0069 | 0.0041 | 0.0566 | 0.0094 | 0.0833 |
| 309 | 0.0226 | 0.0120 | 0.0043 | 0.0331 | 0.0084 | 0.0943 |
| 310 | 0.0168 | 0.0067 | 0.0043 | 0.0584 | 0.0095 | 0.0879 |
| 311 | 0.0194 | 0.0103 | 0.0038 | 0.0390 | 0.0086 | 0.0943 |
| 312 | 0.0184 | 0.0089 | 0.0044 | 0.0464 | 0.0088 | 0.0891 |
| 313 | 0.0238 | 0.0119 | 0.0040 | 0.0310 | 0.0083 | 0.0870 |
| 314 | 0.0194 | 0.0101 | 0.0040 | 0.0400 | 0.0086 | 0.0877 |
| 315 | 0.0193 | 0.0100 | 0.0044 | 0.0415 | 0.0087 | 0.0870 |
| 316 | 0.0165 | 0.0062 | 0.0043 | 0.0603 | 0.0097 | 0.0789 |
| 317 | 0.0213 | 0.0112 | 0.0040 | 0.0351 | 0.0084 | 0.0907 |
| 318 | 0.0162 | 0.0058 | 0.0043 | 0.0651 | 0.0100 | 0.0890 |
| 319 | 0.0187 | 0.0092 | 0.0042 | 0.0442 | 0.0087 | 0.0874 |
| 320 | 0.0435 | 0.0119 | 0.0042 | 0.0204 | 0.0083 | 0.0892 |
| 321 | 0.0187 | 0.0092 | 0.0045 | 0.0452 | 0.0088 | 0.0860 |
| 322 | 0.0317 | 0.0131 | 0.0042 | 0.0246 | 0.0083 | 0.0934 |
| 323 | 0.0240 | 0.0121 | 0.0042 | 0.0311 | 0.0083 | 0.0875 |
| 324 | 0.0186 | 0.0094 | 0.0042 | 0.0439 | 0.0088 | 0.0876 |
| 325 | 0.0173 | 0.0081 | 0.0039 | 0.0504 | 0.0091 | 0.0918 |
| 326 | 0.0160 | 0.0054 | 0.0041 | 0.0664 | 0.0102 | 0.0807 |
| 327 | 0.0178 | 0.0083 | 0.0042 | 0.0497 | 0.0090 | 0.0880 |
| 328 | 0.0191 | 0.0100 | 0.0041 | 0.0413 | 0.0087 | 0.0891 |
| 329 | 0.0166 | 0.0065 | 0.0043 | 0.0595 | 0.0096 | 0.0859 |
| 330 | 0.0195 | 0.0102 | 0.0043 | 0.0407 | 0.0087 | 0.0873 |
| 331 | 0.0164 | 0.0064 | 0.0042 | 0.0613 | 0.0097 | 0.0923 |
| 332 | 0.0171 | 0.0077 | 0.0040 | 0.0528 | 0.0092 | 0.0921 |
| 333 | 0.0210 | 0.0112 | 0.0041 | 0.0365 | 0.0085 | 0.0937 |
| 334 | 0.0173 | 0.0076 | 0.0043 | 0.0532 | 0.0093 | 0.0871 |
| 335 | 0.0227 | 0.0116 | 0.0046 | 0.0338 | 0.0084 | 0.0851 |
| 336 | 0.0174 | 0.0077 | 0.0043 | 0.0527 | 0.0092 | 0.0869 |
| 337 | 0.0182 | 0.0092 | 0.0041 | 0.0449 | 0.0088 | 0.0940 |
| 338 | 0.0188 | 0.0095 | 0.0041 | 0.0430 | 0.0087 | 0.0885 |
| 339 | 0.0176 | 0.0081 | 0.0043 | 0.0504 | 0.0091 | 0.0883 |
| 340 | 0.0192 | 0.0096 | 0.0047 | 0.0444 | 0.0087 | 0.0884 |
| 341 | 0.0165 | 0.0066 | 0.0042 | 0.0601 | 0.0096 | 0.0952 |
| 342 | 0.0179 | 0.0081 | 0.0045 | 0.0508 | 0.0091 | 0.0854 |
| 343 | 0.0211 | 0.0114 | 0.0043 | 0.0364 | 0.0085 | 0.0921 |
| 344 | 0.0178 | 0.0090 | 0.0038 | 0.0459 | 0.0089 | 0.0941 |
| 345 | 0.0204 | 0.0108 | 0.0041 | 0.0370 | 0.0085 | 0.0952 |
| 346 | 0.0187 | 0.0089 | 0.0043 | 0.0452 | 0.0088 | 0.0792 |
| 347 | 0.0180 | 0.0088 | 0.0041 | 0.0468 | 0.0089 | 0.0903 |
| 348 | 0.0194 | 0.0100 | 0.0045 | 0.0417 | 0.0087 | 0.0859 |
| 349 | 0.0173 | 0.0079 | 0.0042 | 0.0515 | 0.0092 | 0.0859 |
| 350 | 0.0189 | 0.0096 | 0.0041 | 0.0427 | 0.0087 | 0.0889 |
| 351 | 0.0159 | 0.0055 | 0.0041 | 0.0678 | 0.0102 | 0.0930 |
| 352 | 0.0199 | 0.0105 | 0.0043 | 0.0401 | 0.0086 | 0.0878 |
| 353 | 0.0273 | 0.0128 | 0.0044 | 0.0281 | 0.0082 | 0.0851 |
| 354 | 0.0172 | 0.0069 | 0.0046 | 0.0576 | 0.0095 | 0.0870 |
| 355 | 0.0165 | 0.0058 | 0.0044 | 0.0636 | 0.0099 | 0.0815 |
| 356 | 0.0209 | 0.0111 | 0.0041 | 0.0366 | 0.0085 | 0.0894 |
| 357 | 0.0191 | 0.0100 | 0.0041 | 0.0415 | 0.0087 | 0.0897 |
| 358 | 0.0196 | 0.0104 | 0.0040 | 0.0395 | 0.0086 | 0.0916 |
| 359 | 0.0169 | 0.0073 | 0.0041 | 0.0542 | 0.0093 | 0.0869 |
| 360 | 0.0204 | 0.0110 | 0.0040 | 0.0373 | 0.0085 | 0.0919 |
| 361 | 0.0194 | 0.0096 | 0.0044 | 0.0425 | 0.0087 | 0.0826 |
| 362 | 0.0185 | 0.0095 | 0.0040 | 0.0438 | 0.0088 | 0.0914 |
| 363 | 0.0295 | 0.0126 | 0.0039 | 0.0256 | 0.0083 | 0.0943 |
| 364 | 0.0194 | 0.0101 | 0.0042 | 0.0413 | 0.0087 | 0.0859 |
| 365 | 0.0166 | 0.0065 | 0.0042 | 0.0598 | 0.0096 | 0.0863 |
| 366 | 0.0290 | 0.0126 | 0.0041 | 0.0261 | 0.0083 | 0.0940 |
| 367 | 0.0174 | 0.0081 | 0.0041 | 0.0502 | 0.0091 | 0.0875 |
| 368 | 0.0201 | 0.0107 | 0.0039 | 0.0375 | 0.0085 | 0.0913 |
| 369 | 0.0173 | 0.0078 | 0.0042 | 0.0524 | 0.0092 | 0.0901 |
| 370 | 0.0179 | 0.0087 | 0.0041 | 0.0473 | 0.0089 | 0.0920 |
| 371 | 0.0202 | 0.0106 | 0.0041 | 0.0383 | 0.0085 | 0.0893 |
| 372 | 0.0178 | 0.0088 | 0.0044 | 0.0489 | 0.0091 | 0.0988 |
| 373 | 0.0167 | 0.0064 | 0.0045 | 0.0615 | 0.0098 | 0.0915 |
| 374 | 0.0157 | 0.0046 | 0.0040 | 0.0738 | 0.0108 | 0.0873 |
| 375 | 0.0240 | 0.0124 | 0.0042 | 0.0312 | 0.0083 | 0.0861 |
| 376 | 0.0166 | 0.0063 | 0.0043 | 0.0610 | 0.0097 | 0.0854 |
| 377 | 0.0166 | 0.0072 | 0.0041 | 0.0566 | 0.0095 | 0.0973 |
| 378 | 0.0273 | 0.0126 | 0.0042 | 0.0277 | 0.0082 | 0.0872 |
| 379 | 0.0178 | 0.0091 | 0.0042 | 0.0469 | 0.0090 | 0.1010 |
| 380 | 0.0180 | 0.0087 | 0.0042 | 0.0478 | 0.0090 | 0.0888 |
| 381 | 0.0232 | 0.0119 | 0.0042 | 0.0323 | 0.0083 | 0.0867 |
| 382 | 0.0168 | 0.0070 | 0.0042 | 0.0573 | 0.0095 | 0.0900 |
| 383 | 0.0176 | 0.0082 | 0.0041 | 0.0495 | 0.0090 | 0.0861 |
| 384 | 0.0172 | 0.0066 | 0.0046 | 0.0588 | 0.0095 | 0.0810 |
| 385 | 0.0165 | 0.0054 | 0.0046 | 0.0675 | 0.0101 | 0.0846 |
| 386 | 0.0176 | 0.0086 | 0.0040 | 0.0475 | 0.0090 | 0.0909 |
| 387 | 0.0218 | 0.0114 | 0.0045 | 0.0353 | 0.0084 | 0.0877 |
| 388 | 0.0177 | 0.0077 | 0.0044 | 0.0522 | 0.0091 | 0.0832 |
| 389 | 0.0205 | 0.0108 | 0.0043 | 0.0377 | 0.0085 | 0.0883 |
| 390 | 0.0184 | 0.0096 | 0.0038 | 0.0425 | 0.0087 | 0.0931 |
| 391 | 0.0178 | 0.0087 | 0.0043 | 0.0470 | 0.0090 | 0.0835 |
| 392 | 0.0215 | 0.0115 | 0.0042 | 0.0352 | 0.0084 | 0.0907 |
| 393 | 0.0169 | 0.0067 | 0.0044 | 0.0582 | 0.0095 | 0.0846 |
| 394 | 0.0167 | 0.0068 | 0.0042 | 0.0587 | 0.0095 | 0.0916 |
| 395 | 0.0191 | 0.0102 | 0.0045 | 0.0426 | 0.0088 | 0.1033 |
| 396 | 0.0204 | 0.0108 | 0.0044 | 0.0384 | 0.0086 | 0.0924 |
| 397 | 0.0201 | 0.0106 | 0.0040 | 0.0385 | 0.0085 | 0.0903 |
| 398 | 0.0156 | 0.0047 | 0.0043 | 0.0753 | 0.0110 | 0.0959 |
| 399 | 0.0182 | 0.0092 | 0.0042 | 0.0460 | 0.0089 | 0.0951 |
| 400 | 0.0190 | 0.0099 | 0.0040 | 0.0417 | 0.0087 | 0.0906 |
| 401 | 0.0183 | 0.0095 | 0.0041 | 0.0445 | 0.0089 | 0.0949 |
| 402 | 0.0160 | 0.0058 | 0.0040 | 0.0648 | 0.0100 | 0.0894 |
| 403 | 0.0173 | 0.0079 | 0.0042 | 0.0521 | 0.0092 | 0.0910 |
| 404 | 0.0223 | 0.0120 | 0.0043 | 0.0341 | 0.0084 | 0.0911 |
| 405 | 0.0165 | 0.0067 | 0.0040 | 0.0583 | 0.0096 | 0.0874 |
| 406 | 0.0180 | 0.0081 | 0.0045 | 0.0502 | 0.0090 | 0.0843 |
| 407 | 0.0176 | 0.0087 | 0.0040 | 0.0478 | 0.0090 | 0.0919 |
| 408 | 0.0169 | 0.0074 | 0.0040 | 0.0537 | 0.0093 | 0.0891 |
| 409 | 0.0210 | 0.0112 | 0.0042 | 0.0363 | 0.0085 | 0.0934 |
| 410 | 0.0188 | 0.0099 | 0.0039 | 0.0418 | 0.0087 | 0.0973 |
| 411 | 0.0174 | 0.0079 | 0.0043 | 0.0518 | 0.0092 | 0.0908 |
| 412 | 0.0190 | 0.0098 | 0.0041 | 0.0422 | 0.0087 | 0.0894 |
| 413 | 0.0169 | 0.0072 | 0.0043 | 0.0561 | 0.0094 | 0.0920 |
| 414 | 0.0175 | 0.0085 | 0.0038 | 0.0478 | 0.0090 | 0.0877 |
| 415 | 0.0164 | 0.0063 | 0.0042 | 0.0611 | 0.0097 | 0.0874 |
| 416 | 0.0163 | 0.0067 | 0.0039 | 0.0590 | 0.0096 | 0.0950 |
| 417 | 0.0186 | 0.0095 | 0.0041 | 0.0435 | 0.0088 | 0.0888 |
| 418 | 0.0211 | 0.0114 | 0.0038 | 0.0344 | 0.0084 | 0.1024 |
| 419 | 0.0166 | 0.0063 | 0.0044 | 0.0612 | 0.0098 | 0.0846 |
| 420 | 0.0169 | 0.0069 | 0.0043 | 0.0574 | 0.0095 | 0.0861 |
| 421 | 0.0194 | 0.0097 | 0.0046 | 0.0436 | 0.0087 | 0.0843 |
| 422 | 0.0174 | 0.0083 | 0.0042 | 0.0504 | 0.0091 | 0.0943 |
| 423 | 0.0168 | 0.0074 | 0.0039 | 0.0548 | 0.0093 | 0.0918 |
| 424 | 0.0174 | 0.0082 | 0.0041 | 0.0502 | 0.0091 | 0.0911 |
| 425 | 0.0181 | 0.0086 | 0.0043 | 0.0478 | 0.0090 | 0.0858 |
| 426 | 0.0158 | 0.0048 | 0.0040 | 0.0709 | 0.0104 | 0.0822 |
| 427 | 0.0185 | 0.0093 | 0.0042 | 0.0447 | 0.0088 | 0.0908 |
| 428 | 0.0201 | 0.0107 | 0.0040 | 0.0380 | 0.0085 | 0.0913 |
| 429 | 0.0208 | 0.0111 | 0.0041 | 0.0364 | 0.0085 | 0.0924 |
| 430 | 0.0165 | 0.0061 | 0.0042 | 0.0625 | 0.0097 | 0.0880 |
| 431 | 0.0230 | 0.0119 | 0.0039 | 0.0321 | 0.0083 | 0.0881 |
| 432 | 0.0293 | 0.0126 | 0.0045 | 0.0266 | 0.0081 | 0.0807 |
| 433 | 0.0166 | 0.0064 | 0.0043 | 0.0598 | 0.0097 | 0.0828 |
| 434 | 0.0216 | 0.0111 | 0.0049 | 0.0372 | 0.0084 | 0.0832 |
| 435 | 0.0166 | 0.0071 | 0.0041 | 0.0569 | 0.0095 | 0.0962 |
| 436 | 0.0173 | 0.0076 | 0.0042 | 0.0536 | 0.0092 | 0.0889 |
| 437 | 0.0165 | 0.0060 | 0.0044 | 0.0633 | 0.0099 | 0.0869 |
| 438 | 0.0156 | 0.0043 | 0.0043 | 0.0766 | 0.0111 | 0.0758 |
| 439 | 0.0182 | 0.0092 | 0.0042 | 0.0454 | 0.0088 | 0.0932 |
| 440 | 0.0171 | 0.0079 | 0.0039 | 0.0514 | 0.0091 | 0.0914 |
| 441 | 0.0196 | 0.0099 | 0.0045 | 0.0416 | 0.0086 | 0.0821 |
| 442 | 0.0164 | 0.0068 | 0.0039 | 0.0579 | 0.0095 | 0.0937 |
| 443 | 0.0178 | 0.0089 | 0.0041 | 0.0471 | 0.0090 | 0.0974 |
| 444 | 0.0237 | 0.0118 | 0.0039 | 0.0310 | 0.0083 | 0.0903 |
| 445 | 0.0201 | 0.0105 | 0.0042 | 0.0390 | 0.0086 | 0.0848 |
| 446 | 0.0213 | 0.0112 | 0.0041 | 0.0351 | 0.0085 | 0.0926 |
| 447 | 0.0170 | 0.0079 | 0.0038 | 0.0520 | 0.0092 | 0.0911 |
| 448 | 0.0175 | 0.0083 | 0.0041 | 0.0492 | 0.0090 | 0.0886 |
| 449 | 0.0156 | 0.0043 | 0.0042 | 0.0780 | 0.0111 | 0.0921 |
| 450 | 0.0221 | 0.0118 | 0.0042 | 0.0342 | 0.0084 | 0.0913 |
| 451 | 0.0198 | 0.0103 | 0.0043 | 0.0399 | 0.0086 | 0.0867 |
| 452 | 0.0176 | 0.0083 | 0.0043 | 0.0501 | 0.0091 | 0.0882 |
| 453 | 0.0183 | 0.0091 | 0.0044 | 0.0460 | 0.0089 | 0.0909 |
| 454 | 0.0171 | 0.0078 | 0.0041 | 0.0526 | 0.0092 | 0.0938 |
| 455 | 0.0209 | 0.0110 | 0.0042 | 0.0366 | 0.0084 | 0.0879 |
| 456 | 0.0174 | 0.0074 | 0.0046 | 0.0546 | 0.0093 | 0.0842 |
| 457 | 0.0161 | 0.0050 | 0.0044 | 0.0703 | 0.0104 | 0.0846 |
| 458 | 0.0202 | 0.0101 | 0.0049 | 0.0411 | 0.0086 | 0.0827 |
| 459 | 0.0158 | 0.0053 | 0.0040 | 0.0681 | 0.0103 | 0.0916 |
| 460 | 0.0203 | 0.0107 | 0.0041 | 0.0380 | 0.0085 | 0.0873 |
| 461 | 0.0175 | 0.0082 | 0.0040 | 0.0503 | 0.0091 | 0.0915 |
| 462 | 0.0190 | 0.0097 | 0.0042 | 0.0425 | 0.0087 | 0.0899 |
| 463 | 0.0180 | 0.0089 | 0.0045 | 0.0489 | 0.0091 | 0.1016 |
| 464 | 0.0162 | 0.0062 | 0.0040 | 0.0621 | 0.0098 | 0.0927 |
| 465 | 0.0168 | 0.0072 | 0.0041 | 0.0561 | 0.0094 | 0.0915 |
| 466 | 0.0165 | 0.0069 | 0.0040 | 0.0571 | 0.0095 | 0.0892 |
| 467 | 0.0156 | 0.0046 | 0.0041 | 0.0737 | 0.0108 | 0.0872 |
| 468 | 0.0187 | 0.0092 | 0.0046 | 0.0459 | 0.0088 | 0.0878 |
| 469 | 0.0230 | 0.0116 | 0.0043 | 0.0327 | 0.0083 | 0.0878 |
| 470 | 0.0174 | 0.0054 | 0.0053 | 0.0662 | 0.0100 | 0.0772 |
| 471 | 0.0172 | 0.0080 | 0.0038 | 0.0509 | 0.0091 | 0.0918 |
| 472 | 0.0174 | 0.0084 | 0.0040 | 0.0489 | 0.0090 | 0.0939 |
| 473 | 0.0187 | 0.0092 | 0.0044 | 0.0452 | 0.0088 | 0.0857 |
| 474 | 0.0184 | 0.0095 | 0.0041 | 0.0440 | 0.0088 | 0.0934 |
| 475 | 0.0170 | 0.0071 | 0.0045 | 0.0560 | 0.0095 | 0.0813 |
| 476 | 0.0169 | 0.0070 | 0.0042 | 0.0563 | 0.0094 | 0.0850 |
| 477 | 0.0166 | 0.0071 | 0.0041 | 0.0564 | 0.0095 | 0.0928 |
| 478 | 0.0183 | 0.0091 | 0.0040 | 0.0447 | 0.0088 | 0.0883 |
| 479 | 0.0202 | 0.0108 | 0.0041 | 0.0379 | 0.0085 | 0.0928 |
| 480 | 0.0174 | 0.0085 | 0.0039 | 0.0488 | 0.0090 | 0.0948 |
| 481 | 0.0219 | 0.0116 | 0.0040 | 0.0341 | 0.0084 | 0.0920 |
| 482 | 0.0167 | 0.0071 | 0.0040 | 0.0562 | 0.0094 | 0.0897 |
| 483 | 0.0189 | 0.0098 | 0.0041 | 0.0421 | 0.0087 | 0.0904 |
| 484 | 0.0181 | 0.0088 | 0.0044 | 0.0479 | 0.0090 | 0.0921 |
| 485 | 0.0213 | 0.0109 | 0.0047 | 0.0375 | 0.0084 | 0.0810 |
| 486 | 0.0177 | 0.0083 | 0.0043 | 0.0500 | 0.0091 | 0.0928 |
| 487 | 0.0177 | 0.0085 | 0.0042 | 0.0488 | 0.0090 | 0.0906 |
| 488 | 0.0183 | 0.0086 | 0.0044 | 0.0476 | 0.0089 | 0.0831 |
| 489 | 0.0162 | 0.0058 | 0.0043 | 0.0652 | 0.0100 | 0.0896 |
| 490 | 0.0198 | 0.0109 | 0.0039 | 0.0382 | 0.0086 | 0.1024 |
| 491 | 0.0204 | 0.0106 | 0.0043 | 0.0380 | 0.0085 | 0.0863 |
| 492 | 0.0191 | 0.0100 | 0.0040 | 0.0411 | 0.0086 | 0.0923 |
| 493 | 0.0168 | 0.0070 | 0.0042 | 0.0566 | 0.0094 | 0.0866 |
| 494 | 0.0156 | 0.0046 | 0.0041 | 0.0726 | 0.0107 | 0.0759 |
| 495 | 0.0181 | 0.0088 | 0.0044 | 0.0475 | 0.0090 | 0.0895 |
| 496 | 0.0180 | 0.0088 | 0.0043 | 0.0477 | 0.0090 | 0.0895 |
| 497 | 0.0162 | 0.0064 | 0.0041 | 0.0606 | 0.0098 | 0.0872 |
| 498 | 0.0173 | 0.0073 | 0.0044 | 0.0550 | 0.0094 | 0.0813 |
| 499 | 0.0187 | 0.0089 | 0.0046 | 0.0467 | 0.0089 | 0.0843 |
| 500 | 0.0249 | 0.0124 | 0.0040 | 0.0297 | 0.0083 | 0.0902 |

**Reference**

1. Budiansah I, Hardiansyah D, Riana A, Pawiro SA, Beer AJ, Glatting G. Accuracy and precision analyses of single-time-point dosimetry utilising physiologically-based pharmacokinetic modelling and non-linear mixed-effects modelling. EJNMMI Phys. 2025;12:26. https://doi.org/10.1186/s40658-025-00726-7

2. Gear JI, Cox MG, Gustafsson J, Gleisner KS, Murray I, Glatting G, et al. EANM practical guidance on uncertainty analysis for molecular radiotherapy absorbed dose calculations. Eur J Nucl Med Mol Imaging. 2018;45:2456–74. https://doi.org/10.1007/s00259-018-4136-7

3. Barrett PH, Bell BM, Cobelli C, Golde H, Schumitzky A, Vicini P, et al. SAAM II: Simulation, Analysis, and Modeling Software for tracer and pharmacokinetic studies. Metabolism. 1998;47:484–92. https://doi.org/10.1016/s0026-0495(98)90064-6

4. Perazzolo S. SAAM II: A general mathematical modeling rapid prototyping environment. CPT Pharmacometrics Syst Pharmacol. 2024;13:1088–102. https://doi.org/10.1002/psp4.13181

5. Hardiansyah D, Yousefzadeh-Nowshahr E, Kind F, Beer AJ, Ruf J, Glatting G, et al. Single-Time-Point Renal Dosimetry Using Nonlinear Mixed-Effects Modeling and Population-Based Model Selection in [^177^Lu]Lu-PSMA-617 Therapy. J Nucl Med 2024; 65:566-572; https://doi.org/10.2967/jnumed.123.266268
